# Influence of acculturation on the ERP correlates of self- and other-referencing

**DOI:** 10.3758/s13415-026-01453-x

**Published:** 2026-05-27

**Authors:** Ashley Gilliam, Qihao Xie, Alena Lokhmanenko, Angela Gutchess

**Affiliations:** 1 Brandeis University, Waltham, MA USA; 2 Department of Psychology, Appalachian State University, 100 Smith Wright Hall, 222 Joyce Lawrence Lane, Boone, NC 28608 USA

**Keywords:** Acculturation, Self-reference effect, Memory, Late frontal positivity, Late positive potential, Cognitive strategies

## Abstract

Acculturation, or cultural change in individuals through continuous contact between distinct cultural groups, is an important construct to study to understand the mechanisms behind effects of cultural background, values, and environment on thoughts and behaviors. This study examined the impact of acculturation on cognitive strategy usage, specifically investigating change in self- and other-referencing strategies in memory among Chinese international students as they acculturated to the US. The current study replicated in a new sample behavioral memory effects associated with acculturation. Importantly, it also extended research by examining the relationship between acculturation and ERP components (P300, late frontal positivity, late positive potential) that represent processes associated with memory strategies (self-/other-referencing) that differ across cultures. In a sample of 56 Chinese students studying in the US, we found that greater acculturation to the US was associated with a larger behavioral self-reference effect, contrasted by a smaller neural self-reference effect (late frontal positivity and late positive potential). However, results provide mixed support for cognitive differences with acculturation being due to one’s processing of the self and changes in one’s neural response to the degree of self-relatedness of information. Instead, we posit the potential for level of effortful processing, or the level of effort put forth to engage in a strategy, as a mechanism for culture's influence on memory strategy usage. Future research should explore these associations, including measuring change in EEG/ERP measures longitudinally over the course of the acculturation process.

**Supplementary Information:**

The online version contains supplementary material available at https://doi.org/10.3758/s13415-026-01453-x.

A multitude of international and cross-cultural comparison studies between global Eastern and Western cultures have demonstrated that there is cultural variation in strategies employed for an array of abilities, including perception, attention, memory, and even affect valuation (Han et al., 2013; Ji et al., 2000; Kitayama et al., 2003; Leger & Gutchess, 2021; Masuda & Nisbett, 2001; Wang & Conway, 2004). However, these group comparisons prevalent in the literature fail to capture the influence of cultural mixing and exchange that so often occurs in modern society owing to increasing globalization (Chen et al., 2015a). The present study addresses this gap by focusing on acculturation or cultural change in individuals as a result of continuous contact between distinct cultural groups, as a factor that could influence the use or effectiveness of cognitive strategies. Considering how interactions between individuals and their surrounding environments can lead to change in cognitive strategies over time is important for identifying the mechanisms through which culture affects cognition. In particular, the current study examined self- and other-referencing as mechanisms through which effects of acculturation could affect memory performance.

This approach to studying acculturation is informed by the dynamic constructivist view, in which culture is conceptualized as a changeable whole made up of many moving parts, which exert influence upon—and receive influence from—other cognitive domains, such as perception and memory of our environments (Hirschfeld & Gelman, 1994; Hong et al., 2000). This approach developed from work with bicultural individuals, who can hold multiple cultural identities and perspectives that may seem in opposition to each other. Priming, or bringing to consciousness one cultural identity or another (e.g., for a Chinese American, their Chinese *or* American identity), demonstrates that bicultural individuals can switch frames of mind based on the cultural context that is primed through the language used or icons (e.g., Great Wall of China; Disneyland) displayed (Benet-Martínez et al., 2002; Hong et al., 2000). Priming studies with bicultural individuals demonstrate the malleability of cognitive strategies based on cultural context in individuals who have already acquired multiple cultural identities. Some researchers have begun to adopt a dynamic perspective to investigate cultural influences over time (Chen et al., 2015a; Gilliam & Gutchess, 2025). Rather than emphasizing the outcomes of moment-to-moment activation in stable cultural backgrounds, these studies focused on the process itself of change or learning of culture over time.

We previously found that changes in acculturation orientation were associated with changes in self-referencing and other-referencing strategies in memory over the course of 16 months (Gilliam & Gutchess, 2025). This was the first demonstration in the literature of *cognitive* change associated with an explicit measure of acculturation. Although a great deal of work has examined the effect of acculturation on sense of self, identity, well-being, and social interaction (Chen et al., 2015a, 2015b; Cheung et al., 2011; Chudek et al., 2015; Cramer et al., 2016; Heine & Lehman, 2004), little to no work had previously examined the effect of acculturation on cognition, especially in terms of memory and mnemonic strategies. The effects on memory emerged when investigating acculturative change within an individual; we failed to find effects of acculturation when comparing across individuals statically at one time point (Gilliam & Gutchess, 2024, 2025). However, previous work has shown that even when cultural effects do not appear behaviorally they can be detected neurally (Gutchess et al., 2006; Kitayama & Uskul, 2011). This suggests that neural measures could be a sensitive assay of effects of acculturation across individuals, perhaps reflecting their potential to be used to isolate specific processes (e.g., those associated with self-referencing when multiple processes could contribute to equally high behavioral memory performance). In our previous studies of acculturation’s impact on cognition, we did not measure neural activity. By focusing on neural patterns, the current study may have sensitivity to probe processes underlying the behavioral memory differences associated with acculturation. Specifically, our project evaluated whether acculturation was related to individual differences in behavioral self- and other-referencing and their neural correlates.

As such, a combination of self-report, behavioral, and cognitive neuroscience (ERP) approaches were used to 1) examine which social and cultural acculturative factors are associated with individual differences in the self-reference memory effect in Chinese international students, and 2) what processes associated with self- and other-referencing underlie the associations between acculturation and strategy usage in memory.

## Neural markers of self- and close other-referencing

Self-referencing evokes larger behavioral memory benefits in Westerners than East Asians (Ng & Lai, 2009; Sui et al., 2007; Zhang et al., 2020; Zhu et al., 2007). Using fMRI, corresponding differences in medial prefrontal cortex activity reflect cultural differences (Wagner et al., 2012; Zhu et al., 2007). Using ERP, cultural groups have been shown to differ in their processing of self versus close others (e.g., N200, P300, P200), primarily evidenced in oddball paradigms (Fan et al., 2013; Salvador et al., 2020; Sui & Humphreys, 2015; Varnum & Hampton, 2017; Zhong et al., 2018). For example, Americans show stronger measures of self-enhancement and self-referential processing compared with Chinese and other East Asians (Hampton & Varnum, 2018; Salvador et al., 2020). Prior work implicates both earlier attentional and later semantic processing stages in self-related effects, including regarding culture (Cai et al., 2016; Fan et al., 2013; Fields et al., 2021; Porter et al., 2021). Based on the prior literature, we examined three ERP components (P300, LFP, LPP) as neural measures of self- and close other-referencing.

The P300 has been previously proposed as an index of the degree of the self-reference effect, such that its amplitude is largest for highly self-relevant information (Fan et al., 2013; Gray et al., 2004; Porter et al., 2021). There is also some suggestion in the literature that the P300 represents degree of encoding and specificity, rather than degree of self-relevance itself. For example, Chen et al. (2011) found that larger P300 amplitudes were elicited by the participants' own names, followed by their province, and that there were no differences in P300 amplitudes between country names (China and USA). Thus, in our study, the P300 was used as a neural marker of level of self-relevance and tested for associations with acculturation. The P300 offers the additional benefit of being an early attentional ERP measure, allowing us to consider whether cultural effects are more likely to be due to attention or semantic processing.

The late positive potential (LPP) and late frontal positivity (LFP) component both signify sustained attention and elaborative processing, and they are both elicited by self-referential information (Fields et al., 2021; Porter et al., 2021). Participants’ names or faces (Tacikowski & Nowicka, 2010), self-relevant words (Fields et al., 2021; Gray et al., 2004), and self-relevant objects (Miyakoshi et al., 2007) elicit greater posterior positivity. Late frontal effects for self-referential information, as indexed with the LFP, have been shown in a number of studies, including with memory, but it is still debated whether this is unique to self-related information or due to generally deeper processing of information (Kotlewska & Nowicka, 2016; Magno & Allan, 2007; Mao et al., 2017; Nowicka et al., 2018; Porter et al., 2021). The LPP, a more parietally distributed ERP component, is more commonly studied than the related LFP; however, there is mixed evidence regarding the extent to which the more posterior effects of the LPP are elicited for self-relevant information, potentially due to stimulus features and task design (Porter et al., 2021). In some prior work, the LPP was examined alongside and disambiguated from the more frontally distributed LFP based on electrode site (Kissler & Hauswald, 2022; Righi et al., 2014). Both the LFP and LPP were examined in this study, allowing us to differentiate anterior and posterior scalp distributions for late positive effects for any potential acculturative associations with self-related and other-related memory encoding.

Although thought to underlie similar processes, certain aspects of task design can lead to stronger effects in either more frontal (LFP) or parietal (LPP) areas (see self-referencing and emotional valence studies: Cunningham et al., 2005; Dolcos & Cabeza, 2002; Fabiani et al., 1990; Fields, 2023; Forester et al., 2020; Karis et al., 1984; Porter et al., 2021; Watts et al., 2014). Both components were examined in the current study to fully capture effects and potentially differentiate between frontal and parietal late positive effects considering cross-study variation in their strength according to scalp distribution.

These three ERP components (LFP, LPP, P300) were used to examine how acculturation affects the self-reference effect in memory during acculturation. Specifically, we tested whether neural measures of self- and close other-referencing were associated with acculturation levels. We expected that these ERP components would index the degree to which participants engaged with self- and other-referencing of information. Based on previous research demonstrating that Americans engage in self-referencing more than Chinese individuals (Huff et al., 2013; Zhu et al., 2007), we predicted that Chinese international students who self-reported greater acculturation to the US would exhibit larger neural self-reference effects (higher amplitudes for LFP, LPP, and P300) than Chinese international students who self-reported less acculturation to the US. Essentially, greater acculturation to the United States would include more self-focus typical of American culture and values. Greater self-focus is expected to then lead to processing self-related information more deeply during encoding, which would also increase memory performance for information related to the self, in contrast to information related to others.

In this study, close other-referencing (a memory benefit for information related to someone with whom one has a close relationship) will be examined alongside self-referencing for a few important reasons. First, our prior work demonstrated that, among Chinese international students in the US, changes in both self- and close other-referencing are associated with acculturative change (Gilliam & Gutchess, 2025). Second, there is a growing scientific literature suggesting that there is a close other-reference effect that uniquely boosts memory for Chinese populations, although this does not appear to extend to distant others and has been primarily investigated for parental figures (Qi & Zhu, 2002; Song et al., 2012; Sui et al., 2007). Lastly, likely due to the predominant focus on Western samples in cognition (Barrett, 2020; Gutchess & Rajaram, 2023; Henrich et al., 2010), this close other-reference effect has been studied much less and is thus less understood than that of self-referencing. Neural indexes of degree of self-relevance described below are expected to not only capture self-referencing effects, but also close other-referencing effects, due to the potential integration of close others into one’s sense of self thought to be associated with East Asian cultures (e.g., as evidenced with fMRI in Zhu et al., 2007).

## Acculturation

We defined acculturation as culture change in individuals through continuous contact between distinct cultural groups, consistent with our prior work and “acculturative strategy” as defined by Berry (1992; Gilliam & Gutchess, 2024, 2025). Our prior research found that changes in one explicit measure of acculturation and in one explicit measure of cultural values were associated with change in memory strategy usage (Gilliam & Gutchess, 2025). These measures were adjusted host acculturation orientation, or how acculturated one reported being to the host (US) culture relative to their home (Chinese) culture, and adjusted independence, or how much one endorsed an independent relative to an interdependent self-construal, respectively. More specifically, adjusted host acculturation orientation captured the desire to engage in one’s home culture (Chinese) traditions and behaviors and maintain relationships with its group members, versus the desire to engage in one’s host culture (US) traditions and behaviors and form relationships with its group members. Adjusted independence captured a focus on one’s relationships, duties, and the needs of others (more typical of dominant Chinese culture) versus a focus on one’s self, achievements, and one’s own goals (more typical of dominant US culture).

Whereas our prior behavioral work identified that these two measures were associated with acculturative change in cognitive strategy usage, for the current study, we were interested in more fully capturing the process of acculturation to the US for a Chinese international student population. As such, several measures were administered that capture unique aspects of the acculturative process for our sample, Chinese international students studying abroad in the US.^1^ Measures included aspects of acculturation and relevant cultural values used by other scholars in social and cultural psychology studies, including sociocultural adaptation, psychological adaptation, conformity, tradition, and perceived cultural distance (Demes & Geeraert, 2014; Galchenko & Van De Vijver, 2007; Güngör, 2007; Roccas et al., 2000; Searle & Ward, 1990; Schwartz, 1992). In order to maximize statistical power, acculturation to the US was operationalized in our current study via a composite acculturation measure. We conducted an exploratory factor analysis in order to create a composite measure of acculturation valid for Chinese international students studying abroad in the US.

## Hypothesis

We hypothesized that there would be acculturative differences in self- and other-referencing as indicated by behavioral memory performance (H1), such that.

- **H1a)** Participants who are *more* acculturated to the US will have a larger behavioral *self-reference* effect in memory (i.e., remember more words that were judged relative to the self than words judged relative to another person). This pattern would reflect the stronger orientation towards the host (US) cultural environment, which emphasizes self-focused values.
- **H1b)** Participants who are *less* acculturated to the USA will have a larger *other-reference* effect in memory (i.e., remember more words that were judged relative to a close other relative to words judged in a control condition). This pattern would reflect the stronger orientation towards home (Chinese) culture environment, which emphasizes other-focused values.

We also hypothesized that there would be acculturative differences in self- and other-referencing as indicated by ERP measures of strategy usage (H2), such that.

- **H2a–c)** Participants who are *more* acculturated to the US will have larger neural responses to the *self* compared with a close other (greater neural self-reference effect), assessed by the LFP (a), LPP (b), and P300 (c). These patterns would reflect a stronger orientation towards the American host culture environment and its self-focused values.
- **H2d–f)** Participants who are *less* acculturated to the US will have larger neural responses to a *close other* compared with a farm animal control condition (greater close-reference effect), assessed by the LFP (d), LPP (b), and P300 (c). These patterns would reflect a stronger orientation towards the Chinese home culture environment and its other-focused values.

Hypotheses and associated statistical analyses were pre-registered using the Preregistration for Quantitative Research in Psychology (PRP-QUANT) template and can be found on OSF.io (https://osf.io/dvwcp/overview?view_only=abb08b6571c944638e4693bdb7e86060).

## Methods

### Participants

Sixty-five Chinese Brandeis University students aged 18–30 years were recruited for the experiment involving EEG and behavioral components. A power analysis suggested that a sample size of 52 was needed for pre-registered linear regression analysis based on a medium effect size and power of 0.8. Although our focus is on analyses at time one, we recruited 65 Chinese, assuming there would be exclusions and possible attrition for a sample at a second time point. Note that due to high attrition rates (~ 50%) and likely underpowered analyses, all results from the second time point of data collection were included as a supplemental material only (see supplemental F).

Participants were recruited using a psychology participant pool, printed flyers placed around the Brandeis University campus, and advertisements posted on Chinese-language social media apps (i.e., WeChat). To be eligible, participants must have identified as originally being from China (we did not restrict based on region), have been in the US for less than 5 years,^2^ and not have had a history of head trauma or seizure.

Where possible, participant data was retained for behavioral analyses despite later exclusions from neural analyses due to EEG artifacts. Four participants were excluded due to either 1) technical issues during data collection or 2) missing responses for substantial portions of scales important for the acculturation composite measure. Only one participant was removed due to poor memory performance (overall memory performance below chance). This means the final behavioral analysis sample size was 60 participants. See Table 1 for demographics of the complete behavioral sample. Details of the neural sample (n = 53 at time point one) after artifact exclusions were made can be found in supplemental A.

**Table 1:** Table of demographics and predictor for behavioral sample

Demographic variable	T1
	*n* = *60*
Age	19.55 (1.57)
Gender	
Female	38.33%
Male	30.00%
*Missing/unanswered (technical issues)*	*31.67%*
Initial time in the US at T1 (months)	12.47 (14.46)
TOEFL score	105.33 (5.95)
Proportion of friends who are Chinese	
A few of them	1.67%
About half of them	13.33%
All of them	25%
Most of them	60%
Adjusted independence *(Independence minus interdependence)*	0.24 (0.9)
Adjusted host score *(Host minus home acculturation orientation)*	− 0.23 (1.25)
Sociocultural adaptation	4.83 (0.95)
Psychological adaptation	4.62 (0.89)
Perceived cultural distance	5.2 (0.88)
Conformity	− 3.29 (2.18)
Tradition	− 1.95 (1.97)

Table presents demographics for full behavioral sample. See supplemental A for a complementary table focused on the neural sample, as well as the results of t-tests comparing the two samples. Acculturation composite measure raw items (prior to z-score transformation and exploratory factor analysis) are reported here individually, including scores for adjusted independence, adjusted host, sociocultural adaptation, psychological adaptation, perceived cultural distance, conformity, and tradition. Statistics reported as *M* (*SD*) or percentage

Note that there were technical issues preventing collection of gender identity for some of the sample (Table 1). Follow-up *t*-tests and replicated linear regression model results suggest, this issue did not impact study results (see Supplemental Material I).

#### Materials and procedures

The reported study was approved by the human subjects review committee at Brandeis University. Prior to beginning study sessions, informed consent was obtained from all participants.

During encoding, participants viewed 120 trait words (e.g., smart) on a computer screen and indicated with a keypress (yes/no) whether the word describes, across trials, themselves, a close other, or a farm animal. “Farm animal” was chosen as a distant other reference, because category norms for farm animal were equivalent for Chinese and American participants (Yoon et al., 2004). Participants all experienced three self-referential memory conditions (self, close other, and farm animal); to equate emotion across the conditions, three levels of emotional valence (positive, negative, and neutral) were balanced across each condition. Prior to beginning the encoding task, participants were asked to choose a “close other” (one person) and “farm animal” (one category like “pigs” or “cows” in general) to think of throughout the duration of the experiment (as in Gilliam & Gutchess, 2024, 2025; Zhang et al., 2020).

Word lists were the same as in our prior self-referencing studies (Gilliam & Gutchess, 2024, 2025). Emotional valence was accounted for using likability ratings of words (e.g., “mean” has a lower likability rating than “wise”) from a prior study (Anderson, 1968; previously used in a Taiwanese sample in Zhang et al., 2020). Similar numbers of low, medium, and high likeability words were assigned to three lists to achieve similar average likability ratings across word lists. These lists were then assigned in a counterbalanced manner to the self, close other, and farm animal conditions across participants. See details regarding list item translations for the experimental task in Gilliam & Gutchess, 2024. Word items (adjectives like “friendly” or “rude”) were presented in subject-specific random order for 4 s each at encoding with 1,500–1,750 ms intervals between targets (ITI was randomly jittered to avoid target expectancy) while EEG data were recorded.

There was a 10-min break during which the EEG cap was removed. After the break, participants returned for an incidental memory retrieval task. During retrieval, participants decided whether each item presented on screen was either “old” (previously studied) or “new” (not studied previously), responding with a keypress. No EEG data were recorded during retrieval.

EEG was collected continuously during encoding using a BioSemi ActiveTwo EEG system with 32 Ag/AgCl electrodes in an elastic cap and the ActiView EEG acquisition software. Electrodes were placed according to the international 10–20 system. Voltage offsets were kept within the recommended range (± 40 kΩ). Additional external electrodes were used to monitor for blinks and eye movements, and as the reference (on each mastoid). Data processing was done by using MATLAB coding integrated with EEGLAB and ERPLAB functions, focusing on the P300, LPP, and LFP components to compare self-versus-close other and close other-versus-farm animal (neutral control) during trait judgments at encoding.

The task and scales were presented in Mandarin. Translations and back-translations were conducted by native Mandarin speakers (see more details in Gilliam & Gutchess, 2024). Participants completed questionnaires about demographics and English language skills. Participants also completed the Center for Epidemiologic Studies Depression Scale (CES-D; Zhang et al., 2011) as a measure of depressive symptoms for use as a control variable and several acculturation-related measures to address our primary construct of interest, as described below. Number of depressive symptoms was included as a control variable, because it could potentially impact both self-reference and memory performance (see Wisco, 2009 and Dillon & Pizzagalli, 2018 for related reviews).

##### Acculturation construct

Acculturation was operationalized as a composite scale which was created by running an exploratory factor analysis on all possible acculturation-related items and extracting factor scores for those that loaded onto one common factor of acculturation. Items included in the EFA to create the composite acculturation measure were based on prior results of an EFA with the same population (see supplemental materials of Gilliam & Gutchess, 2024). Results of this EFA can be found in supplemental E. Final scale items listed below were run in a new exploratory factor analysis for the current study sample and then factor scores were extracted, thus creating a composite acculturation score for each participant.

These factor scores were extracted from the following composite measure items:

1. *The Brief Acculturation Orientation Scale* assessed individuals’ relationship to their culture of origin and relationship to the culture of contact (Demes & Geeraert, 2014). We used an *adjusted host acculturation orientation score*, which was a measure of host orientation relative to home orientation (host minus home), as in our prior studies (Gilliam & Gutchess, 2024, 2025).
2. *The Singelis Self-Construal Scale* assessed how individuals perceive themselves in relation to other individuals, or self-construal (Singelis, 1994). We used an *adjusted independence score*, which was a measure of independence relative to interdependence (independence minus interdependence), as in prior studies (Gilliam & Gutchess, 2024, 2025; Kraus et al., 2021; Yu et al., 2021).
3. *The Schwartz Culture Value Scale* examined cross-cultural attitudes, values, and beliefs, and we extracted from it the dimensions of *conformity* and *tradition*. These subscales were reverse-scored in our study so that higher scores indicated less identification with the values of conformity and tradition (Schwartz, 1992).
4. The *Brief Psychological Adaptation Scale* assessed how comfortable and happy participants felt with respect to being in the new host culture (US), reverse scoring items assessing negative feelings associated with adaptation (e.g., anxious, out of place) (Demes & Geeraert, 2014).
5. The *Brief Sociocultural Adaptation Scale* assessed more practical and behavioral aspects of adapting to a new host culture, such as socioeconomic adaptation challenges, with a higher score indicating easier adaptation (Demes & Geeraert, 2014).
6. The *Brief Perceived Cultural Distance Scale* evaluated the extent to which home culture and host culture were similar, with a higher score indicating greater perceived similarity between the US and China (Demes & Geeraert, 2014).

Final acculturation composite scores used for analyses were moderate in reliability (Cronbach’s alpha = 0.72).

##### Time 2

At the second time point, participants went through the exact same process, but with a new wordlist to avoid familiarity effects in the memory task. This allowed us to assess changes in acculturation or memory strategies over time. However, no EEG data were collected. Details regarding this data and its supplementary analysis can be found in supplemental F.

##### Behavioral memory measure

Overall memory performance across conditions was scored as hits, or a proportion of correct responses to old items, minus false alarms, a proportion of new items mistakenly remembered as being seen before to all possible new items. For all linear regressions, memory performance was quantified using only the hit rate for each reference condition. This is because the false-alarm rate was the same for each condition (i.e., there was only one pool of “new” items rather than being specific to each condition) and thus does not add additional information. Difference scores were calculated for self minus close other, and difference scores were calculated for close minus farm animal.

##### ERP data processing and analysis

Following behavioral data exclusions, seven additional participants were excluded due to an excessive number of artifacts (i.e., based on prior research, our large sample size, and the expected robust difference waves, a criterion was set of at least 5 trials in each self-relevance condition: self, close other, farm animal; Boudewyn et al., 2018). Thus, a total of 53 participants from the initial cohort (N = 65) were included for the ERP analyses.

For ERP analysis, the raw EEG data (recording sampling rate of 2,048 Hz) were recorded without reference. Data were referenced offline to the average of electrodes at the left and right mastoids. Preprocessing and analyses were performed in EEGLAB (https://sccn.ucsd.edu/eeglab/index.php; Delorme & Makeig, 2004) and ERPLAB (https://erpinfo.org/erplab; Lopez-Calderon & Luck, 2014) in MATLAB 2023a. Testing with an oscilloscope demonstrated a negligible delay (less than ~ 20 ms) between the event marker recorded by the EEG system and stimulus presentation, so no event marker shifts were necessary. Segments of data from breaks in the task (longer than 7 s between event codes) were automatically removed. For each period of continuous EEG data, the DC offset was removed by subtracting the average voltage for the segment and filtered using a second-order Butterworth infinite impulse response (IIR) high-pass filter with a half-amplitude cutoff at 0.1 Hz (12 dB/oct roll-off; Kappenman & Luck, 2010). No low pass filter was applied. Data were then downsampled at 512 Hz to facilitate speed of data processing. Then, periods of exceptionally noisy EEG (e.g., extreme voltage offsets, large muscle artifacts) were identified and removed via semi-automatic ERPLAB procedures (as used in Kappenman et al., 2021).

Next, independent components analysis (ICA) was used to correct for ocular artifacts. All EEG channels were first manually inspected for any potential bad channels in need of removal; Independent components were calculated from remaining segments using the infomax runica algorithm in EEGLAB.

The ICA solution was applied to the continuous data. ICLabel was used to automatically classify components. All components were visually inspected, but if components were flagged as 80% or higher probability of being an artifact (e.g., eye, muscle) by ICLabel, they were inspected further for potential removal. There was a range of 0 to 10 components removed with a mean of 3.21 components (SD = 2.4).

Data were epoched to include all trials and baseline periods. Baseline was calculated from the prestimulus window (− 200 ms to 0 ms) and subtracted from the post-stimulus window (0 to 1,000 ms). Afterwards, channels previously marked for interpolation based on visual inspection of the raw data were interpolated using EEGLAB (no interpolated channels were included for ERP measurement and analysis). Then, segments of data containing artifacts that survived the correction procedure were flagged and excluded from analysis using automated ERPLAB procedures with individualized thresholds set based on visual inspection of each participant's data (Luck, 2014; Kappenman et al., 2021). This included excluding trials with large changes in voltage for any channel.

ERPs were calculated separately for each reference condition (self-judgment, other-judgment and farm-judgment). On average, there were 26.79 trials in the close other condition, 26.36 trials in the farm animal condition, and 24.68 trials in the self condition (with a range of 5 to 44 per condition). These conditions were then compared using difference waves (self-other). Difference waves were used in this study as they were particularly useful for isolating the precise variation in magnitude for processes of interest (e.g., level of self-relevance; see further discussions of difference waves: Kappenman & Luck, 2012; Luck, 2014). Mean amplitude of the difference waves across the time windows and electrode clusters detailed below were used for analysis. Multi-site cluster analysis, alongside a priori defined sites and time windows, was used as it reduces the number of statistical comparisons and thus the risk of type 1 error, and it has been shown previously to be equal to or better than single electrode site analyses (Luck & Gaspelin, 2017; Zhang & Kappenman, 2024).

###### The P300 component

**Fig. 1:**
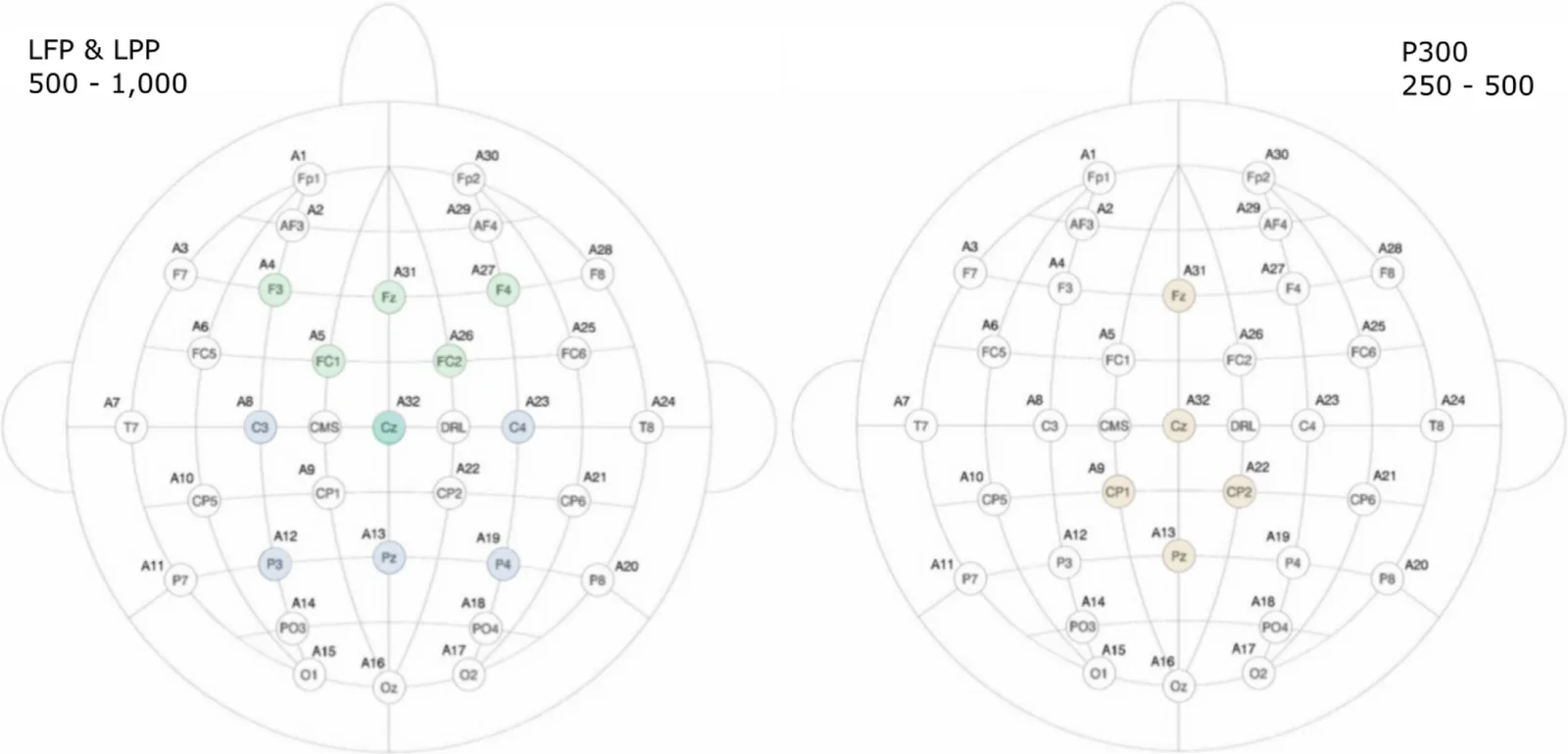
Electrode sites included in clusters for three ERP components of interest. *Note.* From left to right: LFP (green) and LPP (blue), P300 (orange). Electrode sites for LFP included Fz, Cz, F3, F4, FC1, FC2. Mean amplitude for the LFP cluster of electrodes was averaged across the 500–950 ms time window. Electrode sites for LPP included Cz, Pz, C3, C4, P3, P4. Mean amplitude for the LPP cluster of electrodes was averaged across the 500–950 ms time window. Electrode sites for P300 included Fz, Cz, Pz, CP1, CP2. Mean amplitude for the P300 cluster of electrodes was averaged across the 250–500 ms time window

The P300 has been previously demonstrated to be an index of the degree of self-relevance and self-referencing, such that its amplitude is largest for highly self-relevant information (Berlad & Pratt, 1995; Fan et al., 2013; Porter et al., 2021; Shi et al., 2011; Tacikowski & Nowicka, 2010). Based on existing literature with similar paradigms, we defined the P300 component as the most positive peak after the N100-P200-N200 complex in the 250–500 ms poststimulus time window (Conroy & Polich, 2007; Gray et al., 2004). Analysis focused on five primarily centroparietal electrodes (Fz, Cz, Pz, CP1, CP2; Fig. 1). The electrode sites and time windows were chosen based on prior literature establishing standard P300 electrode configurations (Fabiani et al., 1986; Johnson, 1993; Polich, 2007) and the examination of the aggregated grand average from trials (AGAT, or the simple mean of all trials across all conditions and participants), which is an unbiased method of parameter selection that allowed for identification of a positive deflection at around 250–500 ms poststimulus (Brooks et al., 2017; Luck & Gaspelin, 2017).

A larger P300 amplitude in the self condition compared with close other condition at encoding would indicate greater attention to or processing of self-relevant information (Fan et al., 2013). A larger P300 amplitude in the close other condition compared to farm animal condition at encoding would similarly indicate greater attention to or processing of information closer to one’s self.

Next, difference waves were created by subtracting the waveforms for close other from those for self (self-referencing) and by subtracting the waveforms for farm animal from those for close other (close-other referencing). The resulting values were tested for associations with acculturation measures and memory performance.

###### The LFP and LPP components

The late frontal positivity (LFP) and late positive potential (LPP) components have both been shown to signify sustained attention and elaborative processing, and are elicited by self-referential information (Fields et al., 2021; Porter et al., 2021). Based on existing literature with similar paradigms (Porter et al., 2021), we defined the late frontal positivity component and late positive potential during the 500–1,000 ms poststimulus time window. Scalp location, defined via electrode site clusters, distinguish the two components. For the LFP, we focused on six midline and frontal electrodes (Fz, Cz, FC1, FC2, F3, F4; Fig. 1), because these have previously been shown to be important for measuring the LFP and distinguishing it from other effects, such as the P300 (Nogueira et al., 2015; Porter et al., 2021; Woźniak et al., 2018). For the LPP, we focused on six central and parietal electrodes (Cz, Pz, C3, C4, P3, P4; Fig. 1) based on prior literature (Cai et al., 2016; Fields et al., 2021). Furthermore, the selection of the 500–1,000 ms time window was supported by examining the aggregated grand average from trials (AGAT, or the simple mean of all trials across all conditions and participants), which is an unbiased method of parameter selection that allowed for identification of a positive deflection at around 500–1,000 ms poststimulus (Brooks et al., 2017; Luck & Gaspelin, 2017).

A larger late positivity, both if distributed more frontally (LFP) or posteriorly (LPP), for the self condition than the close other condition would indicate a larger self-reference effect (SRE), or greater semantic processing of self-relevant information. Similarly, a larger late positivity for the close other condition compared with the neutral control condition, farm animal, would indicate a larger close other-reference effect (ORE), or greater semantic processing of information closer to one’s self.

Analyses mirrored those of the P300. Difference waves were used to test associations between self-referencing and close-other referencing with acculturation measures and memory performance.

###### ERP data quality

Point-wise Standardized Measurement Error (SME) was averaged across the time windows of interest for each ERP cluster (LFP, LPP, P300) per condition in order to evaluate the variability of our ERP measures. Overall, these averages of point-wise SME by bin (self, close, and farm animal condition individually) were within a typical range ($\widehat{SEM}$ = 0.67 to 1.14) with an overall average, collapsing across bins, of 0.87 (Zhang & Luck, 2023). This indicates our neural measures used in analysis were derived from standard quality, low noise data.

#### Analytic plan

Data was analyzed using R. The *lm.beta* package was used to extract standardized betas and the *BayesFactor* package was used to extract Bayes factors from linear regression model results.

##### Overall check

An ANOVA was initially used in order to evaluate whether self-referencing and close other-referencing benefits were present. Specifically. a repeated measures one-way ANOVA was run for the full behavioral sample, comparing corrected memory performance (hit rate, percentage of correct recognition of old items, minus false-alarm rate, percentage of incorrect responses to new items irrespective of condition) by reference condition (3 within-subject levels: self, close other, and farm animal).

In order to evaluate whether self- and close other-referencing neural patterns were present, exploratory *t*-tests were run for ERP components of interest, comparing by reference condition. This was completed for both overall clusters and individual electrode sites with an FDR adjustment applied to correct for multiple comparisons.

##### Behavioral analysis plan

To address hypothesis one, we tested if *(H1a)* participants who were *more* acculturated to the US would have a larger *self-reference* effect in memory (greater memory for self compared to close other). We then tested if *(H1b)* participants who were *less* acculturated to the US would have a larger *close other-reference* effect in memory (greater memory for close others compared with a neutral control [farm animal]). To test Hypothesis 1a and 1b, we examined the effects of our acculturation composite measure on behavioral measures of memory using the linear regression models. Models were run separately for self- and close other-referencing. Memory performance was measured as hit rate.

*Behavioral models included:*

- *H1a) *Behavioral self-referencing effect in memory:
  $$\begin{array}{c}{BehavSelfRef}_{i}=\alpha +{{\boldsymbol{\beta}}AccultComp}_{{\boldsymbol{i}}}+\varepsilon \\ null: {\beta AccultComp}_{i}=0\end{array}$$
- *H1b) *Behavioral close-referencing effect in memory:
  $$\begin{array}{c}{BehavCloseRef}_{i}=\alpha +{{\boldsymbol{\beta}}AccultComp}_{{\boldsymbol{i}}}+\varepsilon \\ null: {\beta AccultComp}_{i}=0\end{array}$$

*Note:* Here*, i* represents individual participants. Parameter of interest for each hypothesis is bolded.

Behavioral self-referencing in memory (*BehavSelfRef*) was a difference score, created by subtracting memory performance in the close other condition from memory performance in the self condition. Behavioral close other-referencing in memory (BehavCloseRef) was also a difference score, created by subtracting memory performance in the neutral control (farm animal) condition from memory performance in the close other condition.

##### Neural analysis plan

To address hypothesis 2, we tested if participants who were *more* acculturated to the US would have a larger neural *self-reference* effect as indexed by mean amplitude for three ERP markers (*(H2a) Late Frontal Positivity, or LFP, (H2b) Late Positive Potential, or LPP, and (H2c) P300*). We similarly tested if participants who were *less* acculturated to the US would have a larger neural *close other-reference* effect as indexed by three ERP markers (*(H2d) LFP, (H2e) LPP, and (H2f) P300*).

ERPs were calculated separately for each reference condition (self-judgment, close other-judgment, and farm-judgment). These conditions were then compared by using difference waves (self-other, other-farm). Neural indexes were defined using the difference between mean amplitude during prespecified poststimulus time windows for self versus close other condition (self-referencing) and close other versus farm animal condition (close other-referencing) (*see the blue difference waves within their respective time windows in *Fig. 3). Separate linear regression models were used with each ERP index as the outcome and acculturation composite measure as predictor.

*Neural (ERP) models included:*

- *H2a,b,c)* LFP, LPP, & P300 amplitude as index for self-referencing (SRE)
  $$\begin{array}{c}{ERP\_SRE}_{i}=\alpha +{{\boldsymbol{\beta}}AccultComp}_{{\boldsymbol{i}}}+\varepsilon \\ null: {\beta AccultComp}_{i}=0\end{array}$$
- *H2d,e,f)* LFP, LPP, & P300 amplitude as index for close other-referencing (ORE)
  $$\begin{array}{c}ERP\_{ORE}_{i}=\alpha +{{\boldsymbol{\beta}}AccultComp}_{{\boldsymbol{i}}}+\varepsilon \\ null: {\beta AccultComp}_{i}=0\end{array}$$

*Note:* Here, *i* represents individual participants. Parameter of interest for each hypothesis is bolded.

All linear regression models, ERP component cluster sites, and ERP component time windows were pre-registered (https://osf.io/dvwcp/overview?view_only=abb08b6571c944638e4693bdb7e86060).

Supplementary analysis was completed for time point two acculturation. See supplemental F for further details.

## Results

### ANOVA results: Presence of behavioral SRE and ORE

**Fig. 2:**
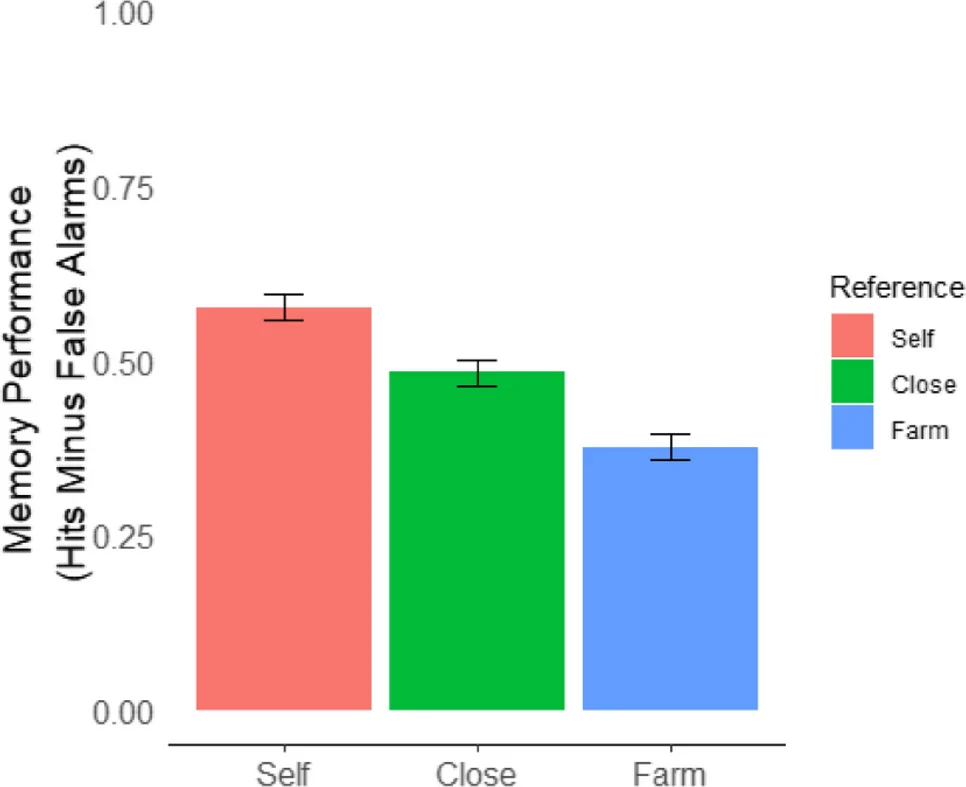
Memory performance by reference condition

A repeated measures one-way ANOVA was run, comparing corrected memory performance (hit rate [percentage of correct recognition of old items] minus false-alarm rate [percentage of incorrect responses to new items, irrespective of condition]) by reference condition. This allowed us to test if self significantly differed from close other and if close other significantly differed from farm animal, thus indicating a behavioral self-reference effect and close other-reference effect (H1a & H1b). See Fig. 2 for a bar graph displaying corrected memory performance by reference condition. Results suggested that there was a significant main effect of reference condition on memory performance (*F*(1.49, 41.79) = 16.20, *p* < 0.001, ηp^2^ = 0.37).^3^

To break down the main effect of reference condition, post-hoc paired *t*-tests with *p*-values adjusted using False Discovery Rate (Benjamini–Hochberg FDR) corrections were used. Memory performance in the self condition was significantly higher than for close other (*t*(63) = 9.89, *p* < .001), which was significantly higher than for farm animal (*t*(63) = 7.45, *p* < .001). This suggests that there were both self- and other-referencing effects in this sample (H1a and H1b). Self condition memory was also significantly better than that for farm animal (*t*(63) = 13.31, *p* < .001).

### t-tests for ERP waveforms by condition: Presence of neural SRE & ORE

**Fig. 3:**
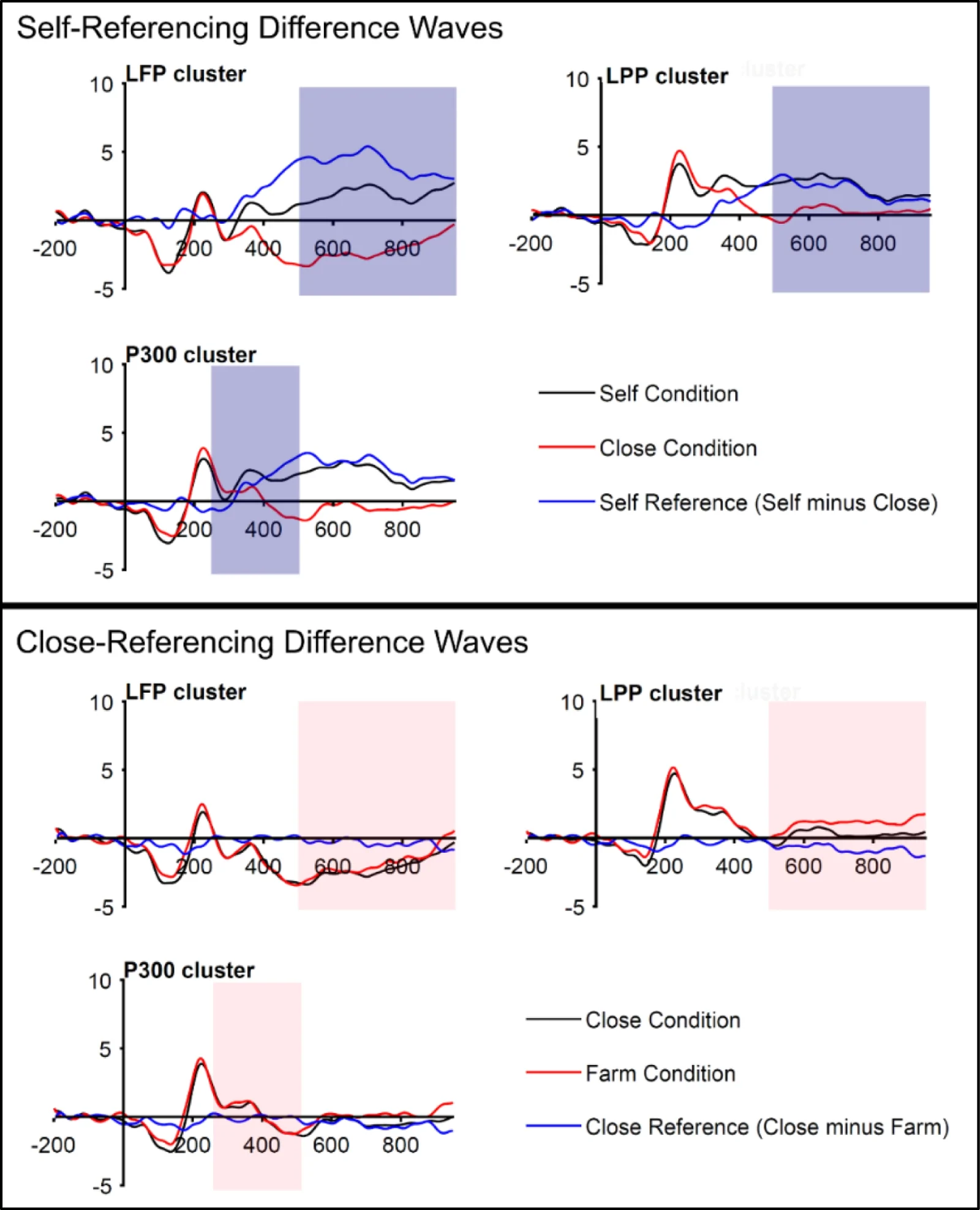
ERP cluster difference waves for self- and close-referencing. *Note.* Figure represents the ERP waveforms for self, close, and farm conditions as well as difference scores for self-referencing and close-referencing. Highlighted areas represent time windows of focus for a priori-defined analyses, across which an average amplitude was extracted (500–1,000 ms for the LFP and LPP; 250–500 ms for the P300). See text for description of included electrodes

To examine if ERP correlates demonstrated the presence of neural self- and other-referencing, exploratory *t*-tests were run comparing amplitude by condition (e.g., self versus close and close versus farm). This information is useful for interpreting regression model results to follow, in that it is possible that there may be a lack of neural self- or close other-referencing effects, but that neural patterns may still relate to acculturation. See Fig. 3 for a visualization of ERP component waveforms by condition.

Results of *t*-tests by condition are in Table 2. *t*-tests were run with directionality specified based on overall cluster visual patterns (Fig. 3). *P*-values were then adjusted for multiple comparisons using an FDR correction. Results suggest that neural self-referencing as indicated by the LFP component (*t*(52) = 5.22, *p* < 0.001) and LPP component (*t*(52) = 3.15, *p* < 0.001) may be more robust than that of P300 (*t*(52) = 2.49, *p* = 0.02). Visually, neural close other-referencing appeared to be in the opposite direction of the expected pattern, such that farm animal (neutral control) exhibited greater positivity than close other. Furthermore, the components identified for close other-referencing were not significant (*p*s > 0.22). As post-hoc tests, individual electrode sites for each component’s cluster were tested as well (Table 2).

**Table 2:** Table of *t*-test comparisons for neural measure amplitude by condition

Condition	Component	Individual electrode sites					
Self vs. Close	P300 *	Fz **	Cz *	Pz	CP1	CP2	
	*t*(52) = 2.49, *p* = 0.02	*t*(52) = 3.50, *p* = 0.002	*t*(52) = 2.46, *p* = 0.02	*t*(52) = 1.38, *p* = 0.15	*t*(52) = 2.07, *p* = 0.06	*t*(52) = 2.03, *p* = 0.06	
	LFP ***	Fz ***	Cz ***	F3 ***	F4 ***	FC1 ***	FC2 ***
	*t*(52) = 5.22, *p* = 0.00002	*t*(52) = 5.45, *p* = 0.00001	*t*(52) = 3.88, *p* = 0.0006	*t*(52) = 4.16, *p* = 0.0003	*t*(52) = 5.98, *p* = 0.000004	*t*(52) = 4.91, *p* = 0.00004	*t*(52) = 4.92, *p* = 0.00004
	LPP **	Cz ***	**Pz**	C3 ***	C4 ***	**P3**	**P4**
	*t*(52) = 3.15, *p* = 0.005	*t*(52) = 3.88, *p* = 0.0006	*t*(52) = 1.44, *p* = 0.15	*t*(52) = 4.16, *p* = 0.0003	*t*(52) = 4.44, *p* = 0.0002	*t*(52) = 1.39, *p* = 0.15	*t*(52) = 1.96, *p* = 0.07
**Close vs. Farm**	**P300**	**Fz**	**Cz**	**Pz**	**CP1**	**CP2**	
	*t*(52) = 0.16, *p* = 0.49	*t*(52) = 0.22, *p* = 0.49	*t*(52) = 0.21, *p* = 0.60	*t*(52) = 0.59, *p* = 0.39	*t*(52) = 0.11, *p* = 0.49	*t*(52) = 0.05, *p* = 0.50	
	**LFP**	**Fz**	**Cz**	**F3**	**F4**	**FC1**	**FC2**
	*t*(52) = 0.38, *p* = 0.46	*t*(52) = 0.55, *p* = 0.39	*t*(52) = 0.11, *p* = 0.49	*t*(52) = 0.75, *p* = 0.77	*t*(52) = 1.43, *p* = 0.15	*t*(52) = 0.26, *p* = 0.49	*t*(52) = 0.68, *p* = 0.36
	**LPP**	**Cz**	**Pz**	**C3**	**C4**	**P3**	**P4**
	*t*(52) = 1.11, *p* = 0.22	*t*(52) = 0.11, *p* = 0.49	*t*(52) = 1.24, *p* = 0.18	*t*(52) = 0.84, *p* = 0.30	*t*(52) = 0.83, *p* = 0.30	*t*(52) = 1.45, *p* = 0.15	*t*(52) = 1.70, *p* = 0.11

*** *p* < 0.001; ** *p* < 0.01; * *p* < 0.05; ꞏ Nonsignificant trend, *p* < 0.10

### Regression MODEL RESULTS: Testing hypotheses

#### H1) Acculturation & Behavioral self-/Other-referencing

For hypothesis one, we predicted that greater acculturation to the United States would be associated with a) larger behavioral self-referencing effects in memory (greater difference between self compared with close other) and b) smaller behavioral close-referencing effects in memory (less of a difference between close other and a neutral control).

To test Hypothesis 1, we examined the effects of our acculturation composite measure on behavioral measures of memory using linear regression models. Models then were run with the addition of number of depressive symptoms (CES-D) as a control variable to account for its potential impact on both acculturation and memory. Results, including this control, are reported in supplemental B.

**Fig. 4:**
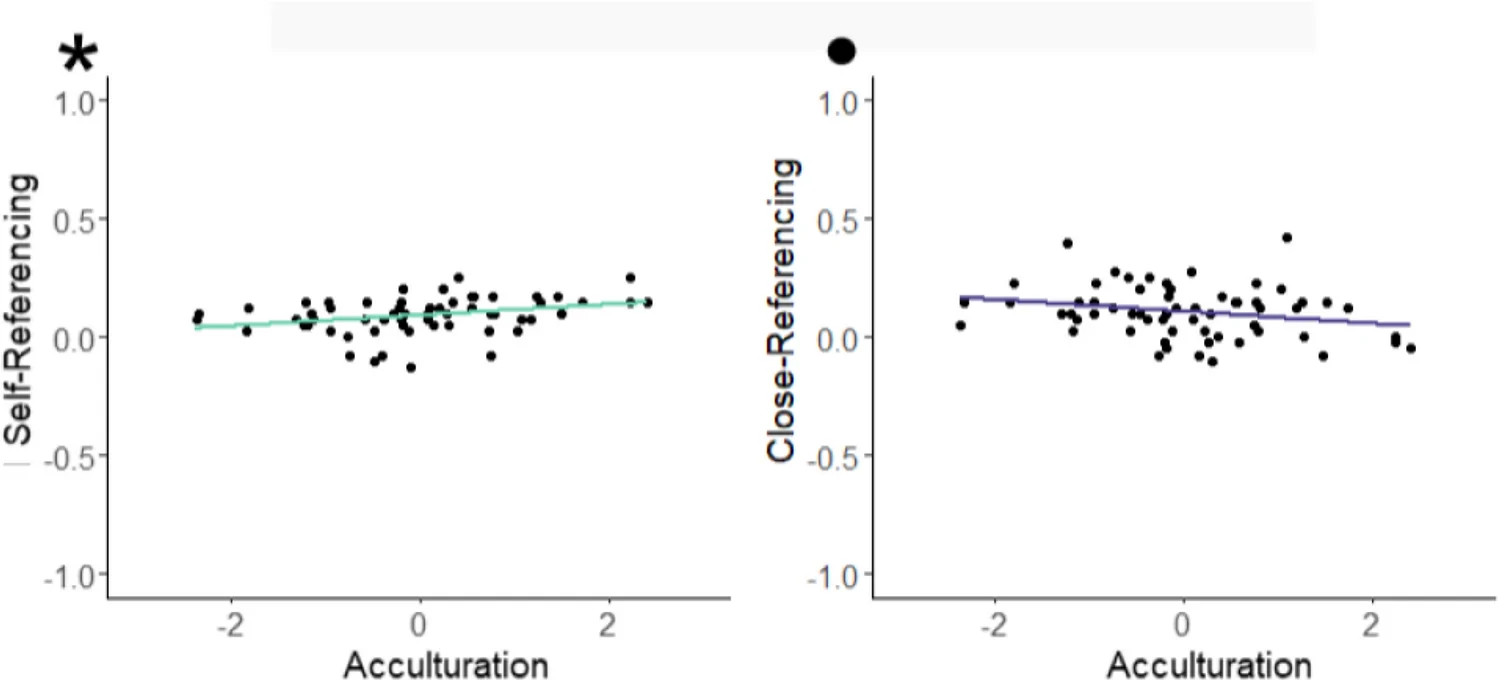
Relationship between acculturation and behavioral memory strategies. *Note.* Results are presented without inclusion of controls. Self-referencing and close other-referencing are represented by difference scores: self minus close other memory and close other minus farm animal (neutral control) respectively. A higher self-referencing score and a higher close other-referencing score indicates greater memory benefits for the self and close others respectively. A higher acculturation composite measure score indicates greater acculturation to the United States (e.g., endorsement of American cultural values, greater ease adapting to the USA). * *p* < 0.05; ꞏNonsignificant trend, *p* < 0.10

Hypothesis one proposed that acculturation might be related to self- and close other-referencing memory strategies. Acculturation was associated with behavioral self-referencing in memory (H1a), such that greater acculturation to the US was associated with higher levels of memory performance for the self versus close other conditions (*t*(59) = 2.52, *p* = 0.01; Fig. 4). This is a medium effect with moderate evidence in favor of our hypothesis (*β* = 0.31, *BF* = 3.52). There was a complementary nonsignificant trend in the expected direction for the effect of acculturation on close-referencing in memory (H1b), such that greater acculturation was associated with less of a difference between memory for close others and a neutral control (farm animals; *t*(59) = − 1.9, *p* = 0.06). This was a small effect with anecdotal evidence in favor of our hypothesis (*β* = − 0.24, *BF* = 1.17).^4^

Results of linear regressions run for behavioral outcomes without those excluded due to neural artifacts (i.e., neural sample) were consistent with those reported in the main text using the full behavioral sample (see Supplemental A).

#### H2) Acculturation and Neural self-/Other-referencing

**Fig. 5:**
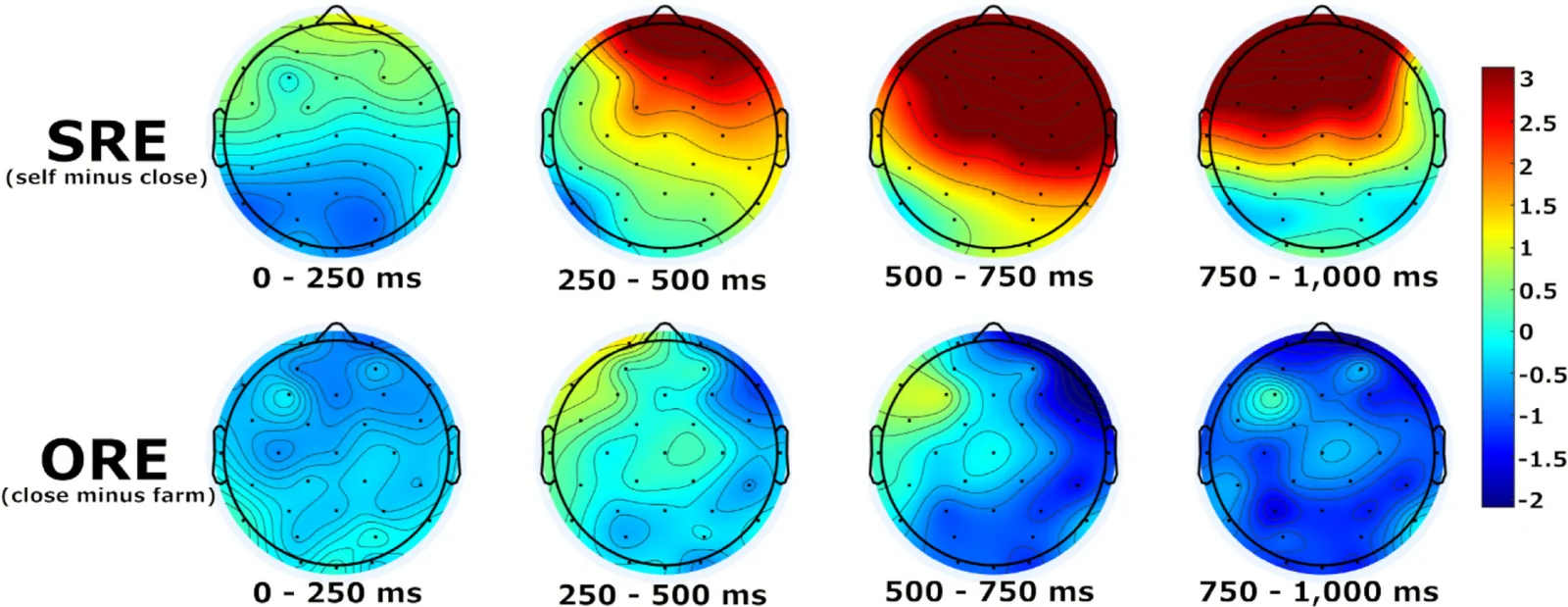
Time series scalp maps self-reference and close other-reference. *Note.* Each scalp map is mean amplitude over a period of 250 ms

For hypothesis 2, we predicted that greater acculturation to the US would be associated with larger self-referencing effects as measured by the a) Late Frontal Positivity (LFP) effect, b) Late Positive Potential (LPP), c) P300, and smaller close-referencing effects d) LFP, e) LPP, f) P300. A time series of scalp maps of mean amplitude at every 250 ms for self- and other-referencing can be found in Fig. 5. Additionally, correlations of each neural measure with corresponding behavioral measures can be found in Supplemental Material B.

To test hypothesis 2, we examined the effects of our acculturation composite measure on self-referencing as measured by the difference between self and other condition mean amplitude for the a) LFP, b) LPP, c) P300, and on close-referencing as measured by the difference between close and farm animal condition mean amplitude for the d) LFP, e) LPP, f) P300 (*see the blue difference waves within their respective time windows in *Fig. 3), using linear regression models.

**Fig. 6:**
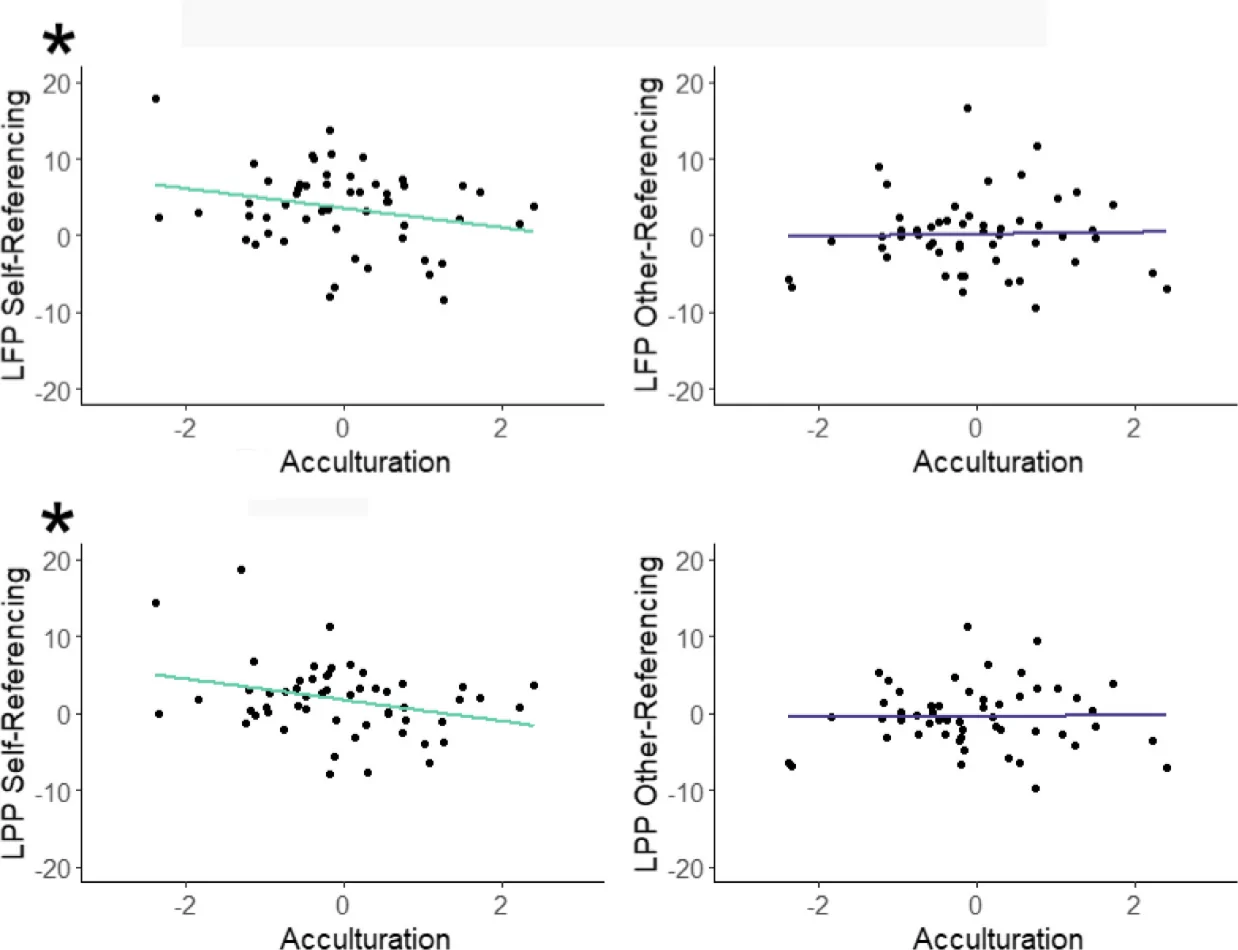
Relationship between acculturation and ERP measures of self- and other-referencing. *Note.* Neural self-referencing and close other-referencing measures are represented by difference scores of mean amplitude (i.e., for the self condition minus close other condition and the close other condition minus the farm animal condition, respectively). These were created separately for the LFP (top row) and LPP (bottom row). A higher neural self-referencing score represents greater processing of self-related information; however, results are opposite of the hypothesized direction. A higher close other-referencing score indicates greater processing of information about close others. A higher acculturation composite measure score indicates greater acculturation to the United States (e.g., endorsement of American cultural values, greater ease adapting to the USA). * *p* < 0.05

In our study, there were significant associations between acculturation and some neural correlates of self-referencing. First, there was a significant association between acculturation and self-referencing as measured by the LFP (H2a), such that greater acculturation was associated with reduced amplitude of the LFP for the self versus close other condition (*t*(52) = − 2.24, *p* = 0.03; Fig. 6). This was a medium effect with anecdotal evidence in favor of the alternative hypothesis (*β* = − 0.3, *BF* = 2.08). Exploratory correlation analyses, testing the effects separately at each electrode, demonstrated that self-referencing as indexed by all the individual LFP sites (Fz, F3, F4, FC1, FC2, and Cz) were significantly negatively correlated with acculturation (see Supplemental D). Scalp maps (Fig. 5) reinforce this finding, with a fairly frontal distribution for neural correlates of self-referencing.

Second, there was a similar significant association between acculturation and self-referencing as measured by the LPP (H2b), such that greater acculturation was associated with reduced amplitude of the LPP for the self versus close other condition (*t*(52) = − 2.28, *p* = 0.03). This was a medium effect with anecdotal evidence in favor of the alternative hypothesis (*β* = − 0.3, *BF* = 2.25). Exploratory correlation analysis of individual LPP sites demonstrated significant negative correlations between self-referencing as indexed by five of six LPP cluster sites (Pz, C3, P3, P4, and Cz) and acculturation (see Supplemental D).

For the P300, acculturation was not significantly associated with the self-referencing (H2c) (*t*(52) = 2.03, *p* = 0.42). This was a small effect with anecdotal evidence for the null hypothesis (*β* = − 0.11, *BF* = 0.36).

Although we find some evidence for relationships between acculturation and self-referencing, the effects of acculturation on close-referencing did not approach significance for any ERP measure (i.e., P300, LFP, LPP; H2d-f: *p*s > 0.43,).

### Discussion

The current study used a combination of behavioral memory, self-report questionnaires, and EEG to evaluate how the acculturation of Chinese international students to the US relates to the neural correlates of self-related cognitive strategies. Results highlight three major patterns. First (*H1a*), higher levels of acculturation to the US were associated with behavioral memory patterns more similar to Americans, meaning greater benefits for self-related information in memory compared to close others. Second (*H1b*), higher levels of acculturation to the US were associated with behavioral memory patterns less similar to Chinese, meaning less of a benefit for close other-related information in memory compared to a neutral control condition. Third (*H2a-c*), our findings for neural correlates of self-referencing were in the opposite direction of our hypotheses and behavioral findings. Specifically, greater acculturation to the US was associated with a reduction in the amplitude difference between self and close, or what might suggest less engagement of neural processes associated with self-relevance and less American-like processing. These three findings will be discussed in more detail below.

### Behavioral findings

The current study replicated our prior findings that acculturation is associated with cognitive strategy usage, and in particular self- and other-referencing memory strategy usage. While our prior behavioral studies (Gilliam & Gutchess, 2024, 2025) emphasized the association in terms of change over time, the current study demonstrated that acculturation is behaviorally related to self- and other-referencing in memory at one time point when comparing across individuals. This association followed a similar pattern to our prior behavioral findings, such that greater self-referencing in memory (*H1a*) and attenuated close other-referencing (*H1b*) in memory was associated with greater acculturation. However, we did not find this association in our prior comparison of the effects of acculturation across individuals (Gilliam & Gutchess, 2024). It is possible that the full acculturation composite measure used in the present study may be more sensitive to effects as compared to acculturation orientation (i.e., to host vs. home culture) alone, as measured in the prior study (Gilliam & Gutchess, 2024). Our results indicate that Chinese international students who are more acculturated to the US engage relatively more in cognitive strategies typical of Americans rather than Chinese. Taken together with the prior literature, these behavioral findings suggest that acculturation might be associated with cognitive strategy usage both within a person as they change over time and in terms of variation between individuals.

### Neural (ERP) findings

Our results suggest that ERP components previously identified as markers of self-relevance and self-referencing effects are associated with acculturative outcomes. However, because the pattern was the opposite of what we had predicted for self-referencing and acculturation, neural findings were mixed in terms of support for our hypotheses. Both the LFP and LPP (*H2b & H2c*) were both significantly associated with acculturation, such that a smaller neural self-reference effect was associated with greater acculturation to the US. This pattern is in stark contrast to our behavioral findings, in which greater acculturation to the US was associated with a larger self-referencing effect in memory. Prior literature has proposed that the LFP and LPP are markers of level of self-relevance; as such, we expected that, similar to behavioral memory for the self, greater acculturation to the US would be associated with deeper processing of self-related information neurally. However, the opposite pattern was present, such that self-referencing as measured by the LFP and LPP decreased with greater acculturation.

Based on this, we propose an alternative explanation of level of effortful processing. This would mean that Chinese international students who were more acculturated to the US more easily engaged in self-referencing strategies because of engagement with American values, including emphasis on the self. This explanation would reflect cultural differences in task difficulty and the concomitant cognitive resources required to complete a task, which has been proposed as one route through which culture can influence cognition (Gutchess et al., 2011). One other aspect of the data that could support this point is the pattern for close other-referencing. There were no associations with acculturation for the LFP, LPP, and P300 as close other-referencing correlates (*H2d, H2e, & H2f*). Yet, in comparisons of ERP component waveforms by condition, close other-referencing visually appeared to be in the opposite direction of the expected pattern. Farm animal displayed a higher amplitude than close other, which may be in line with our proposed explanation of level of effortful processing, such that Chinese international students find it easier to process information in reference to close others than farm animals.

To test whether effortful processing could explain our unexpected findings, we ran post-hoc analyses of reaction time data (supplemental H); however, exploratory findings were not in line with this explanatory framework. Our current study design does not allow for full exploration of this potential underlying mechanism, but future research should further examine it. In addition to significant acculturative effects for the LFP and LPP as indicative of neural self-referencing, we did not see significant associations with acculturation for the P300 as a self-referencing neural correlate (*H2a*).

### Limitations and future directions

One open question is what the window, or sensitive period, for acculturation may be, and how our sample aligns with that. Because the average time in the US for our sample is 1 year, it is possible that several participants were sampled past their peak acculturative period. The sensitive period for acculturation and its effects on cognition has yet to be firmly established, but our prior research found evidence of change within a 16-month period (Cheung et al., 2011; Chudek et al., 2015; Gilliam & Gutchess, 2025). Although simple time spent in the US is an attractive potential measure of acculturation, this factor did not load onto the construct of acculturation in our prior work or relate to acculturative change in memory strategies over time (Gilliam & Gutchess, 2024; see exploratory factor analysis results in supplemental materials). Some work also suggests that instead age at immigration is important for the rate of one’s acculturation, but that this is no sensitive period for acculturation (Cheung et al., 2011; Chudek et al., 2015). Future work should recruit sufficiently large and varied samples to compare effects of acculturation at different time points (e.g., new to the US vs. 1 year in the US) as well as to examine within-individual acculturative effects over time. While not possible in the current study, future work should additionally assess individuals prior to their arrival in a host country; in particular, it would be helpful to understand differences between students who do and do not engage in international studies.

It should be noted that the current study did not explore potential third variables that could interact with culture or acculturative processes. Most studies within cross-cultural psychology focus on international between-culture comparisons, especially in comparing the global East and West. This is also typically done with a particular focus on East Asians and European Americans without consideration of cross-cultural interaction (Gilliam & Gutchess, 2024, 2025; Kitayama & Salvador, 2024). Effect sizes are likely smaller for examinations of acculturation, within-person change, and within-culture regional comparisons as opposed to cross-national comparisons. However, future work in the field could benefit from considering the impact of these potentially modulating variables, including emotion, gender identity, socioeconomic status, age during immigration, and regional variation.

There is a larger literature on self-enhancement and emotion cross-culturally, suggesting that there is a positivity bias for the self-unique to certain contexts (e.g., the Euro-American West and Latin America, in contrast to East Asia; Kitayama, & Rossmaier, 2025; Salvador et al., 2022, 2025), but it typically does not focus on memory (Xie, Gilliam, & Gutchess, *in press*; Zhang et al., 2020). Prior work has found a tendency for Americans to remember more positive information and less negative information with a lack of such an effect for Taiwanese, but it did not find interactions with self-referencing in memory (Zhang et al., 2020). A separate study we conducted using the sample reported here similarly examined potential interactions between acculturation of Chinese international students, level of self-relevance, and emotional valence of items using ERP, but may have been underpowered to detect such effects (Xie et al., *in press*). Future work may benefit from further examining the intersection of self-relevance, memory, and emotion as prior findings are mixed.

In the current study, participants rated their host and home cultures individually, creating an individualized acculturation score based on their personal experience with both countries. However, overall acculturative effects are averaged and may not be reflective of the entirety of either country. Both American and Chinese cultures are heterogenous, although the exact dimensions on which this heterogeneity lies is yet unclear. Little work in the study of cross-cultural psychology has examined within-culture variation, especially within a country and in application to cognition (Conway et al., 2001; Grossmann & Varnum, 2011; Talhelm et al., 2014; Vandello & Cohen, 1999). It is only recently that the field has begun moving beyond broad Eastern/non-Western versus Western comparisons globally to examine differences between areas within a global region (e.g., within non-Western: Arab, Latin American, East Asian, South Asian; Kitayama & Salvador, 2024). Of the newer work that has begun addressing this, the most relevant is that which examines regional variation within China when focusing upon rice-versus-wheat-growing regions (Talhelm et al., 2014). Few studies have examined social status as a form of within-culture variation in cognition well, but recently the field has begun making efforts in this direction with a focus upon socioeconomic status (Grossmann & Varnum, 2011). Issues of rurality/urbanicity, social status, regional identity, and geographic location could be addressed further in future research.

In addition to aspects of within-culture variation, gender and/or sex differences could also interact with cultural effects on cognition. To name a few associated characteristics, societies differ in gender role expectations, whether they are matrilineal or patrilineal, and concepts of femininity and masculinity across the world (Gneezy et al., 2009; Hofstede, 2016; Nanda, 2014). Even within industrialized societies, there is variation in importance placed on sex and gender characteristics. Little work in the cross-cultural cognition field has focused on the intersection with gender. What has been studied provides mixed findings that may reflect small effect sizes (e.g., social orientation; Harb & Smith, 2008; Kashima et al., 1995; Stedham & Yamamura, 2004). With regards to self- and close other-referencing in particular, findings are mixed.^5^ Across studies with both male and female-dominant samples, mother-referencing appears to elicit activation in the same region as self-referencing for Chinese individuals, particularly when integrated with Chinese contextual priming (Ng et al., 2010; Wuyun et al., 2014). In fact, some regions associated with self-related processing may be even more activated for one’s mother than that of the self without any significant gender differences (Huff et al., 2013). However, one study has shown that males in the Chaoshan subculture of China, described as particularly male-dominated, showed only a father-reference effect in contrast to additional self- and mother-reference effects for females (Liu et al., 2017; Song et al., 2012). Future work could benefit from further examining such potential sex and gender differences in understanding acculturation’s impact on cognitive patterns, keeping in mind regional differences in masculinity and patrilineality within both host and home cultural sites.

It should also be noted that it is difficult to tease apart the effects identified here related to the LFP and the LPP. It is possible that the LPP component could generate strong effects that are observable in frontal electrodes despite sources being more posterior (Luck, 2014). In fact, at least one study has found such a pattern (Yang et al., 2019). Although the current study used electrode sites previously shown to distinguish between such components (Kissler & Hauswald, 2022; Righi et al., 2014), future work would benefit from larger electrode arrays that allow for the use of detailed cortical topographic analysis and source localization to distinguish between component effects related to acculturation (Junghöfer et al., 1997; Murray et al., 2008).

## Conclusions

This study contributes to the very small literature demonstrating that acculturation is associated with neural processes involved in cognitive strategy usage. Prior work has focused primarily on the impact of acculturation on social interaction, identity, self-construal (e.g., self-mother overlap; Chen et al., 2015b). Some research suggests that acculturation to the US is related to cognitive performance via neuropsychological tests; however, little to no work has examined strategy usage associated with these cognitive processes (Mendoza et al., 2022; Tan et al., 2021). Our results provide support for the relationship between acculturation and cognitive strategy usage both behaviorally and neurally. We add evidence of the role of *between*-subject variation in acculturation to prior findings (Gilliam & Gutchess, 2025) that *within*-person change in acculturation is associated with memory strategy usage. The most novel contribution from the current study is in demonstrating that neural processes also vary with acculturation. Greater acculturation to the US is associated with a smaller neural self-reference effect (LFP & LPP), a pattern opposite that of the one for behavioral performance and expected from prior literature. Although we speculate that this unexpected pattern could reflect less effortful processing as individuals acculturate to the US and adopt different cognitive strategies, more research is needed. Further investigations of acculturation over time and using multiple measures of brain and behavior will help to elucidate the complex dynamics of acculturation and how these affect cognition.

## Supplementary Information

Below is the link to the electronic supplementary material.

Supplementary file1 (DOCX 167 KB)

## Data Availability

De-identified and averaged data and raw EEG data files (.bdf) are available on OSF.io (https://osf.io/dvwcp/files?view_only=abb08b6571c944638e4693bdb7e86060). Original study materials (not sourced or copyrighted from elsewhere) may be available upon reasonable request. This study was preregistered using the Preregistration for Quantitative Research in Psychology (PRP-QUANT) Template and can be found on OSF.io (https://osf.io/dvwcp/overview?view_only=abb08b6571c944638e4693bdb7e86060).

## Footnotes

1 It should be noted that relevant acculturative measures likely vary depending on the population of interest and domain of cognition being investigated. For example, there are socioemotional differences between German and American cultural groups (Tsai, 2024), but both are high in independent self-construal values (Fernández et al., 2005). In the case of German immigrants to the United States, adjusted independence may not be the most applicable value measure.

2 Note that participants had on average been in the USA for ~ 1 year at the time of participation. There was no significant correlation between scores on the acculturation composite measure and time spent in the United States, (r(58) = − 0.12, *p* = 0.37).

3 Note that degrees of freedom were adjusted for sphericity.

4 This effect reached significance when adding CES-D to the model as a control variable (see Supplemental B).

5 In an analysis of 45 females and 18 males, there was no evidence for gender differences in self-referencing (compared to other referencing), *t*(61) = .29, *p* = .77, or other-referencing (compared with the control condition), *t*(61) = .59, *p* = .56. In order to test this question with a reasonable sample size, we tested this in the sample from Supplement H in Gilliam & Gutchess (2025), which partially overlaps with the sample reported in this manuscript.

## Code availability

Statistical analysis code in R or other research materials are available upon reasonable request.

## Declarations

### Open practices statement

De-identified and averaged data and raw EEG data files (.bdf) are available on OSF.io (https://osf.io/dvwcp/files?view_only=abb08b6571c944638e4693bdb7e86060). Statistical analysis code in R or other research materials are available upon reasonable request. This study was preregistered using the Preregistration for Quantitative Research in Psychology (PRP-QUANT) Template and can be found on OSF.io (https://osf.io/dvwcp/overview?view_only=abb08b6571c944638e4693bdb7e86060).

## References

[CR1] Anderson, N. H. (1968). Likableness ratings of 555 personality-trait words. *Journal of Personality and Social Psychology,**9*(3), 272. 10.1037/h0025907 5666976

[CR2] Barrett, H. C. (2020). Towards a cognitive science of the human: Cross-cultural approaches and their urgency. *Trends in Cognitive Sciences,**24*(8), 620–638. 10.1016/j.tics.2020.05.007 32534836

[CR3] Benet-Martínez, V., Leu, J., Lee, F., & Morris, M. W. (2002). Negotiating biculturalism: Cultural frame switching in biculturals with oppositional versus compatible cultural identities. *Journal of Cross-Cultural Psychology,**33*(5), 492–516.

[CR4] Berlad, I., & Pratt, H. (1995). P300 in response to the subject’s own name. *Electroencephalography and Clinical Neurophysiology/evoked Potentials Section,**96*(5), 472–474. 10.1016/0168-5597(95)00116-a 7555920

[CR5] Berry, A. S., Zanto, T. P., Clapp, W. C., Hardy, J. L., Delahunt, P. B., Mahncke, H. W., & Gazzaley, A. (2010). The influence of perceptual training on working memory in older adults. *PLoS ONE,**5*(7), Article e11537. 10.1371/journal.pone.0011537 PMC2904363 20644719

[CR6] Boudewyn, M. A., Luck, S. J., Farrens, J. L., & Kappenman, E. S. (2018). How many trials does it take to get a significant ERP effect? It depends. *Psychophysiology,**55*(6), Article e13049. 10.1111/psyp.13049 PMC5940511 29266241

[CR7] Brooks, J. L., Zoumpoulaki, A., & Bowman, H. (2017). Data-driven region-of-interest selection without inflating Type I error rate. *Psychophysiology,**54*(1), 100–113. 10.1111/psyp.12682 28000250

[CR8] Cai, H., Wu, L., Shi, Y., Gu, R., & Sedikides, C. (2016). Self-enhancement among Westerners and Easterners: A cultural neuroscience approach. *Social Cognitive and Affective Neuroscience,**11*(10), 1569–1578. 10.1093/scan/nsw072 PMC5040913 27217110

[CR9] Chen, P. H. A., Heatherton, T. F., & Freeman, J. B. (2015). Brain-as-predictor approach: An alternative way to explore acculturation processes. *Neuroscience in Intercultural Contexts*, 143–170.

[CR10] Chen, P. H. A., Wagner, D. D., Kelley, W. M., & Heatherton, T. F. (2015b). Activity in cortical midline structures is modulated by self-construal changes during acculturation. *Culture and Brain,**3*, 39–52. 10.1007/s40167-015-0026-z PMC4520324 26236572

[CR11] Chen, J., Yuan, J., Feng, T., Chen, A., Gu, B., & Li, H. (2011). Temporal features of the degree effect in self-relevance: Neural correlates. *Biological Psychology,**87*(2), 290–295. 10.1016/j.biopsycho.2011.03.012 21470572

[CR12] Cheung, B. Y., Chudek, M., & Heine, S. J. (2011). Evidence for a sensitive period for acculturation: Younger immigrants report acculturating at a faster rate. *Psychological Science,**22*(2), 147–152. 10.1177/0956797610394661 21189354

[CR13] Chudek, M., Cheung, B. Y., & Heine, S. J. (2015). US immigrants’ patterns of acculturation are sensitive to their age, language, and cultural contact but show no evidence of a sensitive window for acculturation. *Journal of Cognition and Culture,**15*(1–2), 174–190.

[CR14] Conroy, M. A., & Polich, J. (2007). Normative variation of P3a and P3b from a large sample: Gender, topography, and response time. *Journal of Psychophysiology,**21*(1), 22–32.

[CR15] Conway, L. G., III., Ryder, A. G., Tweed, R. G., & Sokol, B. W. (2001). Intranational cultural variation: Exploring further implications of collectivism within the United States. *Journal of Cross-Cultural Psychology,**32*(6), 681–697.

[CR16] Cramer, E. S., Dusko, M. J., & Rensink, R. A. (2016). Group-level differences in visual search asymmetry. *Attention, Perception, & Psychophysics,**78*, 1585–1602. 10.3758/s13414-016-1137-0 27270735

[CR17] Cunningham, W. A., Espinet, S. D., DeYoung, C. G., & Zelazo, P. D. (2005). Attitudes to the right-and left: Frontal ERP asymmetries associated with stimulus valence and processing goals. *NeuroImage,**28*(4), 827–834. 10.1016/j.neuroimage.2005.04.044 16039143

[CR18] Delorme, A., & Makeig, S. (2004). EEGLAB: An open source toolbox for analysis of single-trial EEG dynamics including independent component analysis. *Journal of Neuroscience Methods*, *134*(1), 9–21. 10.1016/j.jneumeth.2003.10.009 15102499

[CR19] Demes, K. A., & Geeraert, N. (2014). Measures matter: Scales for adaptation, cultural distance, and acculturation orientation revisited. *Journal of Cross-Cultural Psychology,**45*(1), 91–109.

[CR20] Dillon, D. G., & Pizzagalli, D. A. (2018). Mechanisms of memory disruption in depression. *Trends in Neurosciences,**41*(3), 137–149. 10.1016/j.tins.2017.12.006 PMC5835184 29331265

[CR21] Dolcos, F., & Cabeza, R. (2002). Event-related potentials of emotional memory: Encoding pleasant, unpleasant, and neutral pictures. *Cognitive, Affective, & Behavioral Neuroscience,**2*(3), 252–263. 10.3758/cabn.2.3.252 12775189

[CR22] Fabiani, M., Karis, D., & Donchin, E. (1986). P300 and recall in an incidental memory paradigm. *Psychophysiology,**23*(3), 298–308. 10.1111/j.1469-8986.1986.tb00636.x 3749410

[CR23] Fabiani, M., Karis, D., & Donchin, E. (1990). Effects of mnemonic strategy manipulation in a Von Restorff paradigm. *Electroencephalogry and Clinincal Neurophysiology,**75*(2), 22–35. 10.1016/0013-4694(90)90149-E 1688770

[CR24] Fan, W., Chen, J., Wang, X. Y., Cai, R., Tan, Q., Chen, Y., ..., & Zhong, Y. (2013). Electrophysiological correlation of the degree of self-reference effect. *PLoS ONE,**8*(12), Article e80289. 10.1371/journal.pone.0080289 PMC3846566 24312467

[CR25] Fields, E. C. (2023). The P300, the LPP, context updating, and memory: What is the functional significance of the emotion-related late positive potential? *International Journal of Psychophysiology*. 10.1016/j.ijpsycho.2023.08.005 PMC10838602 37586592

[CR26] Fields, E. C., Bowen, H. J., Daley, R. T., Parisi, K. R., Gutchess, A., & Kensinger, E. A. (2021). An ERP investigation of age differences in the negativity bias for self-relevant and non–self-relevant stimuli. *Neurobiology of Aging,**103*, 1–11. 10.1016/j.neurobiolaging.2021.02.009 PMC8178198 33773473

[CR27] Forester, G., Halbeisen, G., Walther, E., & Kamp, S. M. (2020). Frontal ERP slow waves during memory encoding are associated with affective attitude formation. *International Journal of Psychophysiology,**158*, 389–399. 10.1016/j.ijpsycho.2020.11.003 33181190

[CR28] Galchenko, I., & Van De Vijver, F. J. (2007). The role of perceived cultural distance in the acculturation of exchange students in Russia. *International Journal of Intercultural Relations,**31*(2), 181–197.

[CR29] Gilliam, A. N., & Gutchess, A. (2024). Influence of acculturation and cultural values on the self-reference effect. *Scientific Reports,**14*(1), Article 1624. 10.1038/s41598-023-46210-z PMC10796948 38238341

[CR30] Gilliam, A. N., & Gutchess, A. (2025). Use of self-referencing memory strategies change over time with acculturation. *Cognition,**254*, Article 105985. 10.1016/j.cognition.2024.105985 39426328

[CR31] Gneezy, U., Leonard, K. L., & List, J. A. (2009). Gender differences in competition: Evidence from a matrilineal and a patriarchal society. *Econometrica,**77*(5), 1637–1664.

[CR32] Gray, H. M., Ambady, N., Lowenthal, W. T., & Deldin, P. (2004). P300 as an index of attention to self-relevant stimuli. *Journal of Experimental Social Psychology,**40*(2), 216–224.

[CR33] Grossmann, I., & Varnum, M. E. (2011). Social class, culture, and cognition. *Social Psychological and Personality Science,**2*(1), 81–89.

[CR34] Güngör, D. (2007). The interplay between values, acculturation and adaptation: A study on Turkish-Belgian adolescents. *International Journal of Psychology,**42*(6), 380–392.

[CR35] Gutchess, A., & Rajaram, S. (2023). Consideration of culture in cognition: How we can enrich methodology and theory. *Psychonomic Bulletin & Review,**30*(3), 914–931. 10.3758/s13423-022-02227-5 PMC10258220 36510095

[CR36] Gutchess, A. H., Welsh, R. C., Boduroglu, A., & Park, D. C. (2006). Cultural differences in neural function associated with object processing. *Cognitive, Affective, & Behavioral Neuroscience,**6*(2), 102–109. 10.3758/cabn.6.2.102 17007231

[CR37] Gutchess, A. H., Schwartz, A. J., & Boduroğlu, A. (2011, July). The influence of culture on memory. In *International conference on foundations of augmented cognition* (pp. 67–76). Springer Berlin Heidelberg.

[CR38] Hampton, R. S., & Varnum, M. E. (2018). Do cultures vary in self-enhancement? ERP, behavioral, and self-report evidence. *Social Neuroscience,**13*(5), 566–578. 10.1080/17470919.2017.1361471 28749312

[CR39] Han, S., Northoff, G., Vogeley, K., Wexler, B. E., Kitayama, S., & Varnum, M. E. W. (2013). A cultural neuroscience approach to the biosocial nature of the human brain. *Annual Review of Psychology,**64*(1), 335–359. 10.1146/annurev-psych-071112-054629 22994921

[CR40] Harb, C., & Smith, P. B. (2008). Self-construals across cultures: Beyond independence—interdependence. *Journal of Cross-Cultural Psychology,**39*(2), 178–197.

[CR41] Heine, S. J., & Lehman, D. R. (2004). Move the body, change the self: Acculturative effects on the self-concept. *The Psychological Foundations of Culture,**8*, 305–332.

[CR42] Henrich, J., Heine, S. J., & Norenzayan, A. (2010). The weirdest people in the world? *Behavioral and Brain Sciences,**33*(2–3), 61–83. 10.1017/S0140525X0999152X 20550733

[CR43] Hirschfeld, L. A., & Gelman, S. A. (1994). Toward a topography of mind: An introduction to domain specificity. In *Mapping the mind: Domain specificity in cognition and culture* (pp. 3–35).

[CR44] Hofstede, G. (2016). Masculinity at the national cultural level. In Y. J. Wong & S. R. Wester (Eds.), APA handbook of men and masculinities (pp. 173–186). American Psychological Association. 10.1037/14594-008

[CR45] Hong, Y. Y., Morris, M. W., Chiu, C. Y., & Benet-Martinez, V. (2000). Multicultural minds. A dynamic constructivist approach to culture and cognition. *American Psychologist,**55*(7), 709–720. 10.1037//0003-066x.55.7.709 10916861

[CR46] Huff, S., Yoon, C., Lee, F., Mandadi, A., & Gutchess, A. H. (2013). Self-referential processing and encoding in bicultural individuals. *Culture and Brain,**1*, 16–33.

[CR47] Ji, L. J., Peng, K. P., & Nisbett, R. E. (2000). Culture, control, and perception of relationships in the environment. *Journal of Personality and Social Psychology,**78*(5), 943–955. 10.1037//0022-3514.78.5.943 10821200

[CR48] Johnson, R. A. Y., Jr. (1993). On the neural generators of the P300 component of the event‐related potential. *Psychophysiology,**30*(1), 90–97. 10.1111/j.1469-8986.1993.tb03208.x 8416066

[CR49] Junghöfer, M., Elbert, T., Leiderer, P., Berg, P., & Rockstroh, B. (1997). Mapping EEG-potentials on the surface of the brain: A strategy for uncovering cortical sources. *Brain Topography,**9*(3), 203–217. 10.1007/BF01190389 9104831

[CR50] Kappenman, E. S., & Luck, S. J. (2010). The effects of electrode impedance on data quality and statistical significance in ERP recordings. *Psychophysiology*, *47*(5), 888–904. 10.1111/j.1469-8986.2010.01009.x PMC2902592 20374541

[CR51] Kappenman, E. S., & Luck, S. J. (2012). ERP components: The ups and downs of brainwave recordings. In S. J. Luck, & E. S. Kappenman (Eds.), *The Oxford handbook of event-related potential components* (Vol. 1, pp. 3–30).

[CR52] Kappenman, E. S., Farrens, J. L., Zhang, W., Stewart, A. X., & Luck, S. J. (2021). ERP CORE: An open resource for human event-related potential research. *NeuroImage*, *225*, 117465. 10.1016/j.neuroimage.2020.117465 PMC7909723 33099010

[CR53] Karis, D., Fabiani, M., & Donchin, E. (1984). P300” and memory: Individual differences in the von Restorff effect. *Cognitive Psychology,**16*(2), 177–216.

[CR54] Kashima, Y., Yamaguchi, S., Kim, U., Choi, S. C., Gelfand, M. J., & Yuki, M. (1995). Culture, gender, and self: A perspective from individualism-collectivism research. *Journal of Personality and Social Psychology,**69*(5), 925. 10.1037//0022-3514.69.5.925 7473038

[CR55] Kissler, J., & Hauswald, A. (2022). Different ways to forget: Electrophysiological mechanisms underlying item-method directed forgetting of angry and neutral faces. *Frontiers in Behavioral Neuroscience,**16*, Article 957227. 10.3389/fnbeh.2022.957227 PMC9519987 36187380

[CR56] Kitayama, S., Duffy, S., Kawamura, T., & Larsen, J. T. (2003). Perceiving an object and its context in different cultures: A cultural look at new look. *Psychological Science,**14*(3), 201–206. 10.1111/1467-9280.02432 12741741

[CR57] Kitayama, S., & Rossmaier, A. (2025). Neuro-cultural shaping of self-enhancement motivation. *Advances in Motivation Science, 12*.

[CR58] Kitayama, S., & Salvador, C. E. (2024). Cultural psychology: Beyond east and west. *Annual Review of Psychology,**75*(1), 495–526. 10.1146/annurev-psych-021723-063333 37585666

[CR59] Kitayama, S., Salvador, C. E., Nanakdewa, K., Rossmaier, A., San Martin, A., & Savani, K. (2022). Varieties of interdependence and the emergence of the Modern West: Toward the globalizing of psychology. *American Psychologist,**77*(9), 991. 10.1037/amp0001073 36595393

[CR60] Kitayama, S., & Uskul, A. K. (2011). Culture, mind, and brain: Current evidence and future directions. *Annual Review of Psychology,**62*, 419–449. 10.1146/annurev-psych-120709-145357 21126182

[CR61] Kotlewska, I., & Nowicka, A. (2016). Present-self, past-self and the close-other: Neural correlates of assigning trait adjectives to oneself and others. *European Journal of Neuroscience,**44*(4), 2064–2071. 10.1111/ejn.13293 27285486

[CR62] Kraus, B., Salvador, C. E., Kamikubo, A., Hsiao, N. C., Hu, J. F., Karasawa, M., & Kitayama, S. (2021). Oscillatory alpha power at rest reveals an independent self: A cross-cultural investigation. *Biological Psychology*, *163*, 108118. 10.1016/j.biopsycho.2021.108118 PMC8491569 34019966

[CR63] Leger, K. R., & Gutchess, A. (2021). Cross-cultural differences in memory specificity: Investigation of candidate mechanisms. *Journal of Applied Research in Memory and Cognition,**10*(1), 33–43. 10.1016/j.jarmac.2020.08.016 PMC8136163 34026469

[CR64] Liu, S., Chen, G., Huang, H., Lin, W., Guo, D., Zhao, S., Tian, D., & Su, M. (2017). Patrilineal background of the She minority population from Chaoshan Fenghuang Mountain, an isolated mountain region, in China. *Genomics,**109*(3–4), 284–289. 10.1016/j.ygeno.2017.05.002 28487173

[CR65] Luck, S. J. (2014). *An introduction to the event-related potential technique*. MIT Press.

[CR66] Luck, S. J., & Gaspelin, N. (2017). How to get statistically significant effects in any ERP experiment (and why you shouldn’t). *Psychophysiology,**54*(1), 146–157. 10.1111/psyp.12639 PMC5178877 28000253

[CR67] Luck, S. J., Stewart, A. X., Simmons, A. M., & Rhemtulla, M. (2021). Standardized measurement error: A universal metric of data quality for averaged event‐related potentials. *Psychophysiology,**58*(6), Article e13793. 10.1111/psyp.13793 PMC8169536 33782996

[CR68] Magno, E., & Allan, K. (2007). Self-reference during explicit memory retrieval: An event-related potential analysis. *Psychological Science,**18*(8), 672–677. 10.1111/j.1467-9280.2007.01957.x 17680935

[CR69] Masuda, T., & Nisbett, R. E. (2001). Attending holistically versus analytically: Comparing the context sensitivity of Japanese and Americans. *Journal of Personality and Social Psychology,**81*(5), 922–934. 10.1037/0022-3514.81.5.922 11708567

[CR70] Mao, X., Wang, Y., Wu, Y., & Guo, C. (2017). Self-referential information alleviates retrieval inhibition of directed forgetting effects—An ERP evidence of source memory. *Frontiers in Behavioral Neuroscience,**11*, 187. 10.3389/fnbeh.2017.00187 PMC5641308 29066962

[CR71] Mendoza, L., Garcia, P., Duara, R., Rosselli, M., Loewenstein, D., Greig-Custo, M. T., … Rodriguez, M. J. (2022). The effect of acculturation on cognitive performance among older Hispanics in the United States. *Applied Neuropsychology: Adult, 29*(2), 163–171. 10.1080/23279095.2020.1725888 PMC12108945 32116033

[CR72] Miyakoshi, M., Nomura, M., & Ohira, H. (2007). An ERP study on self-relevant object recognition. *Brain and Cognition,**63*(2), 182–189. 10.1016/j.bandc.2006.12.001 17223240

[CR73] Murray, M. M., Brunet, D., & Michel, C. M. (2008). Topographic ERP analyses: A step-by-step tutorial review. *Brain Topography,**20*(4), 249–264. 10.1007/s10548-008-0054-5 18347966

[CR74] Nanda, S. (2014). Gender diversity: Crosscultural variations. Waveland Press.

[CR75] Ng, S. H., Han, S., Mao, L., & Lai, J. C. (2010). Dynamic bicultural brains: FMRI study of their flexible neural representation of self and significant others in response to culture primes. *Asian Journal of Social Psychology,**13*(2), 83–91.

[CR76] Ng, S. H., & Lai, J. C. L. (2009). Effects of culture priming on the social connectedness of the bicultural self: A self-reference effect approach. *Journal of Cross-Cultural Psychology,**40*(2), 170–186. 10.1177/0022022108328818

[CR77] Nogueira, A. M., Bueno, O. F., Manzano, G. M., Kohn, A. F., & Pompéia, S. (2015). Late positive slow waves as markers of chunking during encoding. *Frontiers in Psychology,**6*, 1032. 10.3389/fpsyg.2015.01032 PMC4516824 26283984

[CR78] Nowicka, M. M., Wójcik, M. J., Kotlewska, I., Bola, M., & Nowicka, A. (2018). The impact of self-esteem on the preferential processing of self-related information: Electrophysiological correlates of explicit self vs. other evaluation. *PLoS ONE,**13*(7), Article e0200604. 10.1371/journal.pone.0200604 PMC6047802 30011309

[CR79] Polich, J. (2007). Updating P300: An integrative theory of P3a and P3b. *Clinical Neurophysiology,**118*(10), 2128–2148. 10.1016/j.clinph.2007.04.019 PMC2715154 17573239

[CR80] Porter, N. A., Fields, E. C., Moore, I. L., & Gutchess, A. (2021). Late frontal positivity effects in self-referential memory: Unique to the self? *Social Neuroscience,**16*(4), 406–422. 10.1080/17470919.2021.1929460 PMC8325518 33978552

[CR81] Qi, J., & Zhu, Y. (2002). The self-reference effect of Chinese college students. *Psychological Science (China)*.

[CR82] Righi, S., Orlando, V., & Marzi, T. (2014). Attractiveness and affordance shape tools neural coding: Insight from ERPs. *International Journal of Psychophysiology,**91*(3), 240–253. 10.1016/j.ijpsycho.2014.01.003 24417862

[CR83] Roccas, S., Horenczyk, G., & Schwartz, S. H. (2000). Acculturation discrepancies and well‐being: The moderating role of conformity. *European Journal of Social Psychology,**30*(3), 323–334.

[CR84] Salvador, C. E., Idrovo Carlier, S., Ishii, K., Torres Castillo, C., Nanakdewa, K., Canale Segovia, F.,…Kitayama, S. (2025). Self-enhancement in Latin America: Is it linked to interdependence? *Personality and Social Psychology Bulletin,* 01461672241309387. 10.1177/01461672241309387 39849676

[CR85] Salvador, C. E., Kamikubo, A., Kraus, B., Hsiao, N. C., Hu, J. F., Karasawa, M., & Kitayama, S. (2022). Self-referential processing accounts for cultural variation in self-enhancement versus criticism: An electrocortical investigation. *Journal of Experimental Psychology: General,**151*(8), 1904. 10.1037/xge0001154 PMC10756494 34807709

[CR86] Salvador, C. E., Kraus, B. T., Ackerman, J. M., Gelfand, M. J., & Kitayama, S. (2020). Interdependent self-construal predicts reduced sensitivity to norms under pathogen threat: An electrocortical investigation. *Biological Psychology,**157*, Article 107970. 10.1016/j.biopsycho.2020.107970 PMC7573572 33096149

[CR87] Schwartz, S. H. (1992). Universals in the content and structure of values: Theoretical advances and empirical tests in 20 countries. *Advances in Experimental Social Psychology/Academic Press*.

[CR88] Searle, W., & Ward, C. (1990). The prediction of psychological and sociocultural adjustment during cross-cultural transitions. *International Journal of Intercultural Relations,**14*(4), 449–464.

[CR89] Shi, Z., Zhou, A., Liu, P., Zhang, P., & Han, W. (2011). An EEG study on the effect of self-relevant possessive pronoun: Self-referential content and first-person perspective. *Neuroscience Letters,**494*(2), 174–179. 10.1016/j.neulet.2011.03.007 21396432

[CR90] Singelis, T. M. (1994). The measurement of independent and interdependent self-construals. *Personality and Social Psychology Bulletin,**20*(5), 580–591.

[CR91] Song, X., Shang, R., Bi, Q., Zhang, X., & Wu, Y. (2012). The influence of sex difference on self-reference effects in a male-dominated culture. *Psychological Reports,**111*(2), 383–392. 10.2466/07.02.10.21.PR0.111.5.383-392 23234084

[CR92] Stedham, Y. E., & Yamamura, J. H. (2004). Measuring national culture: Does gender matter? *Women in Management Review,**19*(5), 233–243.

[CR93] Sui, J., & Humphreys, G. (2015). The integrative self: How self-reference integrates perception and memory. *Trends in Cognitive Sciences,**19*(12), 719–728. 10.1016/j.tics.2015.08.015 26447060

[CR94] Sui, J., Zhu, Y., & Chiu, C.-y. (2007). Bicultural mind, self-construal, and self- and mother-reference effects: Consequences of cultural priming on recognition memory. *Journal of Experimental Social Psychology,**43*(5), 818–824.

[CR95] Tacikowski, P., & Nowicka, A. (2010). Allocation of attention to self-name and self-face: An ERP study. *Biological Psychology,**84*(2), 318–324. 10.1016/j.biopsycho.2010.03.009 20298741

[CR96] Talhelm, T., Zhang, X., Oishi, S., Shimin, C., Duan, D., Lan, X., & Kitayama, S. (2014). Large-scale psychological differences within China explained by rice versus wheat agriculture. *Science,**344*(6184), 603–608. 10.1126/science.1246850 24812395

[CR97] Tan, Y. W., Burgess, G. H., & Green, R. J. (2021). The effects of acculturation on neuropsychological test performance: A systematic literature review. *The Clinical Neuropsychologist,**35*(3), 541–571. 10.1080/13854046.2020.1714740 31996089

[CR98] Vandello, J. A., & Cohen, D. (1999). Patterns of individualism and collectivism across the United States. *Journal of Personality and Social Psychology,**77*(2), 279.

[CR99] Varnum, M. E., & Hampton, R. S. (2017). Cultures differ in the ability to enhance affective neural responses. *Social Neuroscience,**12*(5), 594–603. 10.1080/17470919.2016.1209239 27420406

[CR100] Wagner, D. D., Haxby, J. V., & Heatherton, T. F. (2012). The representation of self and person knowledge in the medial prefrontal cortex. *Wiley Interdiscip Rev Cogn Sciences,**3*(4), 451–470. 10.1002/wcs.1183 PMC3375705 22712038

[CR101] Wang, Q., & Conway, M. A. (2004). The stories we keep: Autobiographical memory in American and Chinese middle-aged adults. *Journla of Pers,**72*(5), 911–938. 10.1111/j.0022-3506.2004.00285.x 15335332

[CR102] Watts, S., Buratto, L. G., Brotherhood, E. V., Barnacle, G. E., & Schaefer, A. (2014). The neural fate of neutral information in emotion‐enhanced memory. *Psychophysiology,**51*(7), 673–684. 10.1111/psyp.12211 24673606

[CR103] Wisco, B. E. (2009). Depressive cognition: Self-reference and depth of processing. *Clinical Psychology Review,**29*(4), 382–392. 10.1016/j.cpr.2009.03.003 19346043

[CR104] Woźniak, M., Kourtis, D., & Knoblich, G. (2018). Prioritization of arbitrary faces associated to self: An EEG study. *PLoS ONE,**13*(1), e0190679. 10.1371/journal.pone.0190679 PMC5749812 29293670

[CR105] Wuyun, G., Shu, M., Cao, Z., Huang, W., Zou, X., Li, S.,…Wu, Y. (2014). Neural representations of the self and the mother for Chinese individuals. *PLoS One, 9*(3), e91556. 10.1371/journal.pone.0091556 PMC3948885 24614597

[CR106] Xie, Q., Gilliam, A., & Gutchess, A. (*In Press*). Impact of Acculturation on Self and Other- Enhancement: N400 & Late Positive Potential. *Psi Chi Journal of Psychological Research*.

[CR107] Yang, H., Laforge, G., Stojanoski, B., Nichols, E. S., McRae, K., & Köhler, S. (2019). Late positive complex in event-related potentials tracks memory signals when they are decision relevant. *Scientific Reports,**9*(1), 9469. 10.1038/s41598-019-45880-y PMC6603184 31263156

[CR108] Yoon, C., Feinberg, F., Hu, P., Gutchess, A. H., Hedden, T., Chen, H. Y. M., ..., & Park, D. C. (2004). Category norms as a function of culture and age: Comparisons of item responses to 105 categories by American and Chinese adults. *Psychology and Aging,**19*(3), 379. 10.1037/0882-7974.19.3.379 15382989

[CR109] Yu, Q., King, A. P., Yoon, C., Liberzon, I., Schaefer, S. M., Davidson, R. J., & Kitayama, S. (2021). Interdependent self-construal predicts increased gray matter volume of scene processing regions in the brain. *Biological Psychology*, *161*, 108050. 10.1016/j.biopsycho.2021.108050 PMC8375393 33592270

[CR110] Zhang, B., Fokkema, M., Cuijpers, P., Li, J., Smits, N., & Beekman, A. (2011). Measurement invariance of the Center for Epidemiological Studies Depression Scale (CES-D) among Chinese and Dutch elderly. *BMC Medical Research Methodology,**11*(1), 1–10. 10.1186/1471-2288-11-74 PMC3123656 21595982

[CR111] Zhang, W., Hung, I. T., Jackson, J. D., Tai, T. L., Goh, J. O., & Gutchess, A. (2020). Influence of culture and age on the self-reference effect. *Aging, Neuropsychology, and Cognition,**27*(3), 370–384. 10.1080/13825585.2019.1620913 PMC6874728 31120367

[CR112] Zhang, W., & Kappenman, E. S. (2024). Maximizing signal-to-noise ratio and statistical power in ERP measurement: Single sites versus multi-site average clusters. *Psychophysiology,**61*(2), Article e14440. 10.1111/psyp.14440 37973199

[CR113] Zhang, G., & Luck, S. J. (2023). Variations in ERP data quality across paradigms, participants, and scoring procedures. *Psychophysiology,**60*(7), Article e14264. 10.1111/psyp.14264 PMC10557079 36748399

[CR114] Zhong, Y., Yin, H., Wang, S., Fan, W., Zhan, Y., & Cai, R. (2018). The self-reference processing of cognitive vulnerability of depression individuals: Evidence from ERPs. *Chinese Science Bulletin,**63*(20), 2026–2035.

[CR115] Zhu, Y., Zhang, L., Fan, J., & Han, S. (2007). Neural basis of cultural influence on self-representation. *NeuroImage,**34*(3), 1310–1316. 10.1016/j.neuroimage.2006.08.047 17134915

