# Supplementary material for: Influence of acculturation on the ERP correlates of self- and other-referencing
Source: Cogn Affect Behav Neurosci. 2026 May 27;26(5):2241–60. doi: 10.3758/s13415-026-01453-x (PMC13614972; doi:10.3758/s13415-026-01453-x)
Supplement: Supplementary file 1 [file 13415_2026_1453_MOESM1_ESM.docx]

**Supplemental Materials**

Influence of Acculturation on the ERP Correlates of Self- and Other-Referencing

Ashley Gilliam^ab^*, Qihao Xie^a^, Alena Lokhmanenko^a^, and Angela Gutchess^a^

^a^Brandeis University, Waltham, MA USA

^b^Appalachian State University, Boone, NC USA

**Supplement A.**

Demographic table, t-test comparisons, and behavioral regression results for neural sample

As detailed in the methods, the sample used for neural analysis was smaller due to electrophysiological (EEG) artifact exclusions. In order to maximize power and avoid the removal of valid data, the behavioral sample retained participants where possible (behavioral memory performance is unaffected by EEG artifacts, e.g, excessive blinking).

A table of demographics for the neural sample can be found below as well the results of Welch two sample t-tests, demonstrating that there are no differences between the samples. Additionally, models were rerun with only neural sample participants to ensure that there was no change in results as compared to results in the main text run with the full behavioral sample. See details below.

**Table A1**

*Table of demographics for neural analysis sample*

| **Demographic variable** | **T1**  *n = 53* |
| --- | --- |
| **Age** | 19.66 (1.62) |
| **Gender**  Female  Male  *Missing/Unanswered due to technical issues* | 37.74%  32.08%  *30.19%* |
| **Time in the USA at T1** (Months) | 12.70 (15.24) |
| **TOEFL Score** | 105.08 (5.92) |
| **Proportion of Friends Who are Chinese**  A few of them  About half of them  All of them  Most of them | 1.89%  15.09%  24.53%  58.49% |
| **Adjusted Independence**  *(Independence minus interdependence)* | 0.26 (0.89) |
| **Adjusted Host Score**  *(Host minus Home acculturation orientation)* | -0.22 (1.31) |
| **Sociocultural Adaptation** | 4.85 (1.00) |
| **Psychological Adaptation** | 4.64 (0.93) |
| **Perceived Cultural Distance** | 2.81 (0.90) |
| **Conformity** | -3.44 (2.10) |
| **Tradition** | -2.02 (1.94) |

There were no differences between behavioral and neural samples in terms of any demographics, memory performance, predictors, or controls. Specifically, the samples used did not differ in terms of age (*t*(56.71) = -0.35, *p* = 0.73), time spent in the United States (months) at time point one (*t*(55.94) = -0.10, *p* = 0.92), TOEFL score (*t*(44.37) = 0.47, *p* = 0.64), overall hit rate (*t*(56.47) = -0.007, *p* = 0.99), overall false alarm rate (*t*(57.46) = 0.34, *p* = 0.74), self condition hit rate (*t*(56.75) = 0.05, *p* = 0.96), close other condition hit rate (*t*(56.59) = -0.06, *p* = 0.95), farm animal condition hit rate (*t*(56.28) = -0.003, *p* = 0.99), acculturation composite score (*t*(57.77) = 0.57, *p* = 0.57), or number of depressive symptoms (*t*(56.27) = -0.25, *p* = 0.80).

Results of linear regressions run for behavioral outcomes without those excluded due to neural artifacts (i.e., neural sample) were consistent with those reported in the main text using the full behavioral sample. Greater acculturation significantly predicted greater self-referencing (t = 2.48, p = 0.02, β = 0.33, BF = 3.25) and did not significantly predict reduced close other-referencing (t = -1.42, p = 0.162, β = -0.20, BF = 0.63). It is worth noting that it is possible that acculturative effects may be stronger for self- as compared to close-other referencing considering the effect sizes reported here and in the main text. This difference in effect sizes could suggest a larger sample size my be needed to determine acculturation’s association with close other-referencing behaviorally.

**Supplement B.**

Analysis Results with Addition of Control Variable

Number of depressive symptoms, as measured by the CES-D (Zhang et al., 2011), was added to all models for time point one presented in the main text (*subsections 2.6.2. and 2.6.3.*). This is because depressive symptoms, including negative affect, low self-worth, feelings of hopelessness, and disturbances of sleep, could affect one’s attitudes, values, and social interaction in an acculturating context as well as affect one’s memory.

Linear regression models for analyses reported below were identical to those of the main text (*subsections 2.6.2. and 2.6.3.*) with the addition of number of depressive symptoms.

***H1) Acculturation & Behavioral Self- / Other-Referencing***

The effect of acculturation on behavioral self-referencing in memory remained significant when adding number of depressive symptoms to the model (β = 0.29, t(58) = 2.38, p = 0.02). The non-significant trend for the effect of acculturation on behavioral close other-referencing in memory reached significance after controlling for number of depressive symptoms (β = -0.26, t(58) = -2.03, p = 0.05).

***H2) Acculturation & Neural Self- / Other-Referencing***

Effects for neural self- and close other-referencing at time point one held after adding our control to the model. When controlling for CES-D, greater acculturation was still significantly associated with reduced amplitude of the LFP (β = -0.31, t(51) = -2.35, p = 0.02) and LPP (β = -0.31, t(51) = -2.34, p = 0.02) for the self versus close other condition (less neural self-referencing).

**Supplement C.**

Correlation Matrix Predictors, Outcomes, & Control Variables

Correlations were run to test for any associations among our predictors, controls, and outcome variables. The neural sample (n = 53 at T1) was used for correlation tests as both neural and behavioral measures were included.

**Figure C1**

Correlation Matrix of Predictors, Outcomes, & Control Variables

In line with our regression findings and hypotheses, acculturation at time point one was significantly positively correlated with behavioral self-referencing at time point one (*r* = 0.33, *p* = 0.02). In the opposite direction of our hypothesis, acculturation at time point one was also significantly negatively correlated with neural self-referencing as measured via the LFP (*r* = -0.31, *p* = 0.02) and LPP (*r* = -0.32, *p* = 0.02). However, there was no association for self-referencing as measured via the P300 (*p* = 0.38).

Acculturation at time point one was not significantly correlated with behavioral (*p* = 0.16) or any measures of neural close other-referencing (*p*s > 0.43)

**Correlations Between Behavioral & Neural Measures of Self- / Other-Referencing**

There was no such association for time point one behavioral self-referencing with any neural self-referencing measures (*p*s > 0.33). No behavioral or neural close other-referencing measures were significantly correlated with one another (*p*s > 0.58).

**Supplement D.**

Details of exploratory correlation analysis of individual electrode sites

Exploratory correlation analysis was run at time point one to test the association between individual electrode sites from clusters for self- and close other-referencing and acculturation composite score. This was to allow better understanding of scalp distribution for cluster effects identified.

**LFP Electrode Sites Correlation with Acculturation**

Exploratory correlation analyses, testing the effects separately at each electrode, demonstrated that self-referencing as indexed by the individual LFP sites of Fz (*r* = -0.30, *p* = 0.03), F3 (*r* = -0.29, *p* = 0.04), F4 (*r* = -0.28, *p* = 0.04), FC1 (*r* = -0.30, *p* = 0.03), FC2 (*r* = -0.32, *p* = 0.02), and Cz (*r* = -0.28, *p* = 0.04) were significantly negatively correlated with acculturation (6 of 6 LFP cluster sites). There were no significant associations between close other-referencing individual electrode sites and acculturation at time point one (*p*s > 0.25).

**LPP Electrode Sites Correlation with Acculturation**

Exploratory correlation analyses, testing the effects separately at each electrode, demonstrated that self-referencing as indexed by the individual LPP sites of Pz (*r* = -0.34, *p* = 0.01), C3 (*r* = -0.34, *p* = 0.01), P3 (*r* = -0.30, *p* = 0.03), P4 (*r* = -0.31, *p* = 0.02), and Cz (*r* = -0.28, *p* = 0.04) were significantly negatively correlated with acculturation (5 of 6 LPP cluster sites). C4 was not significantly correlated with acculturation (*p* = 0.16). There were no significant associations between close other-referencing individual electrode sites and acculturation at time point one (*p*s > 0.18).

**P300 Electrode Sites Correlation with Acculturation**

Exploratory correlation analyses, testing the effects separately at each electrode, found no significant associations between self-referencing as indexed by individual P300 sites and acculturation (0 of 5 P300 cluster sites, *p*s > 0.23). Similarly, there were no significant correlations for close-other referencing as indexed by individual P300 sites (0 of 5 P300 cluster sites, *p*s > 0.28).

**Supplement E.**

Exploratory Factor Analysis Results & Composite Acculturation Measure Details

An exploratory factor analysis (EFA) was performed on questionnaire data from time point one and time point two separately, after which factor scores were extracted for use as our composite acculturation measure.

EFA was completed prior to any data exclusions due to memory performance, artifacts, or technical errors that did not affect the questionnaire completion. Participant exclusions were due to missing data that made it impossible to complete the EFA (i.e., missing most or all of a required scale).

Items included in the EFA were based on the results of our prior EFA in a similar Chinese international student sample (Gilliam & Gutchess, 2024).

The EFA (n = 61 at T1, n = 29 at T2) included seven items:

a. *Adjusted Host Acculturation Score* (host acculturation minus home acculturation; derived from acculturation orientation scale: Demes & Geeraert, 2014)
b. *Adjusted Independence* (independence minus interdependence; derived from self-construal scale: Singelis, 1994)
c. *Sociocultural Adaptation Scale* (Demes & Geeraert, 2014)
d. *Psychological Adaptation Scale* (Demes & Geeraert, 2014)
e. *Conformity* (sub-scale from Schwartz Cultural Value Scale, Schwartz, 1992)
f. *Tradition* (sub-scale from Schwartz Cultural Values Scale, Schwartz, 1992)
g. *Perceived Cultural Distance* (Geeraert, 2014)

Z-scores were used and scores were reversed as necessary so that all scales were coded in the direction of a higher score indicating greater American acculturation (e.g., endorsement of American cultural values, greater ease adapting to the United States). Eigenvalues, scree plots, and parallel analysis were used in combination to determine the number of factors present at both time points.

**Time Point One EFA Results**

Eigenvalues suggested a 3-factor solution was best, the scree plot suggested a 1-factor solution was best, and the parallel analysis suggested a 2-factor solution was best. Considering this, 3, 2, and 1-factor solutions were then examined, testing which best fit the data using a Promax rotation. 3 and 2 factor solutions were not sufficient (p = 0.70, p = 0.46). However, a 1-factor solution was sufficient (X^2^(14, N = 61) = 38.18, *p* < .001).

This 1-factor solution explained 30% of the variance.

Loadings on this common factor included the following:

Conformity	0.89

Tradition	0.91

Adjusted Host score	0.21

Adjusted Independence	0.41

Sociocultural Adaptation	0.17

Psychological Adaptation	0.47

Perceived Cultural Distance	0.17

**Time Point Two EFA Results**

Eigenvalues suggested a 3-factor solution was best, the scree plot suggested a 1-factor solution was best, and the parallel analysis suggested a 1-factor solution was best. Considering this, 3, 2, and 1-factor solutions were then examined, testing which best fit the data using a Promax rotation. A 3-factor solution was not sufficient (p = 0.07). A 2-factor solution was sufficient (X^2^(8, N = 29) = 16.69, *p* = 0.03). A 1-factor solution was also sufficient (X^2^(14, N = 29) = 25.54, *p* = 0.04).

This 1-factor solution explained 30% of the variance.

Loadings on this common factor included the following:

Conformity	0.99

Tradition	0.90

Adjusted Host score	0.25

Adjusted Independence	0.33

Sociocultural Adaptation	0.23

Psychological Adaptation	-0.10

Perceived Cultural Distance	0.19

**Supplemental F**

Time Point Two Behavioral & ERP Analyses

Although we were not able to collect neural data at the second time point due to practical constraints, it was still of theoretical interest to assess whether there was an association between neural correlates of cognitive strategy usage earlier in the process of acculturation (at time one) and acculturation level 7.5 months later (time two). That is, whether patterns of neural activity predicted later processes associated with acculturation. It was also worth exploring whether findings of acculturation at time one replicated at time two. As such, supplemental analyses were performed.

Participants completed two sessions, with the time two follow-up approximately half a year (*M* = 7.26 months, *SD* = 2.70) after the first session. This follow-up period was selected based on previous work that demonstrated change in self-mother distinctions via fMRI for Chinese immigrants with a six-month follow-up (Chen et al., 2015). To minimize attrition, the second time point of data collection was conducted online at a location and time convenient to the participant (i.e., independent of the researcher). Due to reduced sample size, analyses may have been underpowered. See below a demographic table for time point two’s behavioral sample. Due to neural exclusions detailed in the main text (*subsection 2.5*.), the final neural sample size at time point two was 26.

**Table F1**

*Table of Demographics and Predictor for Time Point Two Behavioral Sample*

| **Demographic variable** | **T2**  *n = 28* |
| --- | --- |
| **Age** | 20.11 (1.97) |
| **Gender**  Female  Male | 57.14%  42.86% |
| **Initial Time in The USA at T1** (Months) | 14.70 (15.72) |
| **TOEFL Score** | 104.20 (5.85) |
| **Proportion of Friends Who are Chinese**  A few of them  About half of them  All of them  Most of them | 0.00%  14.29%  21.43%  64.29% |
| **Adjusted Independence**  *(Independence minus interdependence)* | 0.13 (0.82) |
| **Adjusted Host Score**  *(Host minus Home acculturation orientation)* | -0.17 (1.25) |
| **Sociocultural Adaptation** | 4.91 (0.86) |
| **Psychological Adaptation** | 4.54 (1.04) |
| **Perceived Cultural Distance** | 5.31 (0.77) |
| **Conformity** | -2.44 (2.78) |
| **Tradition** | -0.62 (2.90) |

The time two models were structured with neural correlates as outcome measures in order to stay consistent with behavioral and time one neural model structures^[1]^. Models were identical to those of the main text (*subsections 2.6.2. and 2.6.3.*) with the predictor now being time point two acculturation. See models below:

*Behavioral Models included:*

*F.1a)*	Behavioral self-referencing effect in memory:

T2_BehavSelfRef_i_ = α + ***β***T2_AccultComp_i_ + ε

*null:*	*β*T2_AccultComp_i_ = 0

*F.1b)*	Behavioral close-referencing effect in memory:

T2_BehavCloseRef_i_ = α + ***β***T2_AccultComp_i_ + ε

*null:*	*β*T2_AccultComp_i_ = 0

*Neural Models included:*

*F.2a,b,c)* LFP, LPP, & P300 amplitude as index for self-referencing (SRE)

T1_ERP_SRE_i_ = α + ***β***T2_AccultComp_i_ + ε

*null:*	*β*T2_AccultComp_i_ = 0

F.2d,e,f) LFP, LPP, & P300 amplitude as index for close other-referencing (ORE)

T1_LFP_ORE_i_ = α + ***β***T2_AccultComp_i_ + ε

*null:*	*βT2_*AccultComp_i_ = 0

Note that EFA results (e.g., factor loadings, weights) were equivalent between time points for the creation of the primary predictor measure (acculturation composite score; Cronbach’s alpha = 0.56; see supplemental E).

**Linear Regression Results at Time Point Two**

***H1) Acculturation & Behavioral Self- / Other-Referencing***

We then tested to see if there was still an association between acculturation and self and close-other referencing memory strategies about seven months later during the acculturation process. There were no effects of acculturation on behavioral self- or other-referencing in memory at time point two (H1a & b: *p*s > 0.09; *see figure F1*)^[2]^.

**Figure F1**

*Relationship Between Acculturation & Behavioral Memory Strategies at Time Point Two*

*Note.* Results are presented without inclusion of controls. T2 self-referencing represents the difference score of memory performance for the self condition (hit rate, or percentage of correct recognition of old items) minus memory performance for the close other condition at time point two. A higher self-referencing score indicates greater memory benefits for the self. T2 close other-referencing represents the difference score of memory performance for the close other condition (hit rate) minus memory performance for the farm animal (neutral control) condition at time point two. A higher close other-referencing score indicates greater memory benefits for close other. A higher acculturation composite measure score indicates greater acculturation to the United States (e.g., endorsement of American cultural values, greater ease adapting to the USA).

***H2) Acculturation & Neural Self- / Other-Referencing***

Although neural data was only collected at time point one, linear regressions were used to test whether neural patterns of cognitive strategy usage at time point one might be associated with composite acculturation score at a later time point during the acculturative process. That is, whether neural activity at time point one was associated with later acculturation outcomes. Contrasting results at time point one, there were no significant associations between any of our ERP measures of self-referencing at time point one and acculturation measured at time point two (H2a-c : *p*s > 0.70).

However, there were significant associations between acculturation at time point two and some neural correlates of close-referencing. There was a significant association between acculturation at time point two and close-referencing as measured by the LFP at time point one (H2d), such that greater acculturation was associated with less of a difference in amplitude between the close other and neutral control conditions for the LFP (*t*(25) = -2.22, *p* = 0.04; *see figure F2*). This was a medium effect with anecdotal evidence to support our hypothesis (*β* = -0.41, *BF* = 2.04).

**Figure F2**

*Relationship Between Time Point Two Acculturation & ERP Measures of SRE and ORE*

*Note.* Results are presented for time point two without inclusion of controls. Neural self-referencing measures are represented here by the difference score of mean amplitude for the self condition minus mean amplitude for the close other condition, created separately for the LFP and LPP. A higher neural self-referencing score represents greater processing of self-related information, however results are opposite of the hypothesized direction. Neural close other-referencing measures are represented here by the difference score of mean amplitude for the close other condition minus mean amplitude for the farm animal condition, created separately for the LFP and LPP. A higher close other-referencing score indicates greater processing of information closer to the self. A higher acculturation composite measure score indicates greater acculturation to the United States (e.g., endorsement of American cultural values, greater ease adapting to the USA). * indicates p < 0.05.

Similarly, there was a significant association between acculturation at time point two and close-referencing as measured by the LPP at time point one (H2e), such that greater acculturation was associated with less of a difference in amplitude between the close other and neutral control conditions for the LPP (t(25) = -2.44, p = 0.02). This was a medium effect with anecdotal evidence to support our hypothesis (*β* = -0.44, *BF* = 2.84).

As with time point one, acculturation was not significantly associated with close-referencing as measured by the P300 (H2f) (*t*(25) = -1.28, *p* = 0.21). This was a small effect with anecdotal evidence in favor of the null hypothesis (*β* = -0.25, *BF* = 0.65).

**Results at Time Point Two with Addition of Control Variable (CES-D)**

Number of depressive symptoms, as measured by the CES-D (Zhang et al., 2011), was added to all models in order to stay consistent with supplemental material B.

***H1) Acculturation & Behavioral Self- / Other-Referencing***

There were no significant effects of acculturation on behavioral self- and close other-referencing in memory at time point two prior to adding controls. After adding number of depressive symptoms to the model, only the effect of acculturation on behavioral self-referencing in memory displayed a trend in the expected direction; however, it did not reach significance and there was only anecdotal evidence in favor of the null hypothesis (β = -0.32, t(26) = -1.71, p < 0.1, BF = 0.79).

It should be noted that there was a nonsignificant trend for the effect of number of depressive symptoms on close-referencing at time point two, such that greater number of self-reported depressive symptoms was associated with less of a close-reference effect in memory (β = -0.36, t(26) = -1.97, p = 0.06).

***H2) Acculturation & Neural Self- / Other-Referencing***

Effects reported in the main text (*subsection 3.3.2.*) for neural self- and close other-referencing at time point two held after adding our control to the model. When controlling for CES-D, greater acculturation was still significantly associated with reduced amplitude of the LFP (β = -0.43, t(24) = -2.27, p = 0.03) and LPP (β = -0.45, t(24) = -2.46, p = 0.02) for the close other versus neutral control condition (less neural close other-referencing).

**Conclusion**

In contrast to main text findings at time point one (*subsection 3.3.1*), there were no significant associations between acculturation and behavioral memory strategies at time point two. The current study was designed with power in mind for neural analysis at time one. Considering the attrition, small effect sizes, and that there was only anecdotal evidence for the null hypotheses for these tests, the time two analyses were likely underpowered to find behavioral effects at time point two.

We also tested if neural patterns at time point one might be associated with acculturation at time point two. We expected that, as markers of level of self-relevance, that greater neural self-referencing and less neural close other-referencing would be associated with greater acculturation 7.5 months later. Our hypotheses regarding close other-referencing effects and acculturation were partially supported by our findings. This was such that attenuated close other-referencing as measured by the LFP and LPP (H2e & H2f) at time point one was associated with greater acculturation to the US at time point two. These results are both potentially in line with the prior literature’s explanatory framework of level of self-relevance and our proposed mechanism of level of effortful processing (*subsection 4.2*) in terms of the overall pattern with acculturation. However, the difference between close other and farm animal (neutral control condition) was not in the expected direction (*subsections 3.2 and 4.2*), providing more evidence for level of effortful processing.

**Supplemental G**

Variability Plots for Acculturation Composite Measure

It was of interest to examine variability in our primary predictor, acculturation composite score. To address this, a violin plot and histogram were created. Distributions were normal and with variability in initial acculturation composite score.

**Figure G1**

*Variation in Acculturation Composite Measure*

*Note.* Data visualization is for neural sample participants at time point one only (*n* = 53). For panel B, solid line represents mean and dotted lines represent one standard deviation.

**Supplemental H**

Post Hoc Analysis: Reaction Time

Our neural findings unexpectedly demonstrated that acculturation to the United States was associated with a reduced neural self-reference effect, the opposite of what we found in the same sample and prior studies for behavioral self-referencing (Gilliam & Gutchess, 2025). This counterintuitive finding led us to consider that level of self-relevance or depth of processing may not be the underlying mechanism for associations between acculturation and cognitive strategy usage. It may be that using self-referencing as a memory strategy is less demanding to implement for individuals who are more acculturated to the United States. So, we posited a third explanatory factor of *effortful processing*.

We had available reaction time data to try to evaluate this potential explanatory factor; as individuals find a task more demanding and effortful, reaction time should increase. We then ran post hoc analysis of the available reaction time data for time point one, examining whether patterns might reflect level of effort involved in self versus close other-referencing.

First, a one-way ANOVA was run to test if mean reaction time at encoding (at time point one only) varied between reference conditions (self, close other, farm animal). There was a significant main effect of reference condition (F(2, 162) = 4.91, p = 0.009) on mean reaction time. Follow-up Tukey HSD comparisons indicated that the reaction times for the self condition were significantly faster than for the close other condition (p = 0.05) and for the farm animal condition (p = 0.01). However, there was no difference between reaction times in the close other and farm animal condition (p = 0.82). This suggests that referencing the self as a strategy was the easiest for participants, but that it was equally difficult to reference close others as it was to reference farm animals as a strategy during encoding.

**Figure H1**

*Reaction Time by Reference Condition at Time One Encoding*

*Note.* Reaction time is presented here in seconds for easier interpretability.

Next, linear regressions were run to test the potential relationship between acculturation and reaction time. Difference scores were created to isolate self-referencing reaction time (self minus close other) and close other-referencing reaction time (close other minus farm animal).

H.1	RTSelfRef_i_ = α + *β*AccultComp_i_ + ε

H.2	RTSelfRef_i_ = α + *β*AccultComp_i_ + ε

Acculturation composite score did not predict either self-referencing reaction time (*β* = 0.04, *t*(53) = 0.31, *p* = 0.76) or close other-referencing reaction time (*β* = 0.06, *t*(53) = 0.45, *p* = 0.65).

**Supplemental I**

Accounting for Gender

As done for demographics in comparing between neural and behavioral samples, Welch two-sample t-tests were run to address the concern that gender may influence outcome variables of interest. Study design and analyses were not planned to test for gender effects, but this was done to address it as a potential confound.

When comparing participants who were missing gender versus participants who were not, there were no differences behaviorally (self-referencing t(50.13) = 1.41, p = 0.16; close-referencing t(45.05) = -0.37, p = 0.71). Neural and behavioral samples are equivalent (see supplemental material A).

There was also no difference when comparing female and male participants behaviorally (self-referencing t(38.96) = 0.32, p = 0.75; close-referencing t(38.22) = 1.38, p = 0.17), or neurally (LPP Self-referencing t(30.67) = 0.63, p = 0.53; LFP self-referencing t(28.91) = 0.98, p = 0.34) using the appropriate samples. This was run focused on effects found to be significant in the main text.

This was further addressed by re-running significant linear regression models with gender added as a control variable. Our power analysis suggested that a sample size of 52 was needed for pre-registered linear regression analysis based on a medium effect size and power of 0.8. In order to retain this power, regressions were rerun with gender coded as a factor with three levels, ‘female’, ‘male’, and ‘missing’.

Significant effects of acculturation identified in the main text remained significant when gender was added into the model. More specifically, greater acculturation remained associated with greater behavioral self-referencing when controlling for gender (t(56) = 2.71, p = 0.009, β = 0.34, BF = 3.34). Greater acculturation was still associated with less neural self-referencing as indicated by the LFP when controlling for gender (t(49) = -2.16, p = 0.04, β = -0.29, BF = 2.56). Lastly, greater acculturation was still associated with less neural self-referencing as indicated by the LPP when controlling for gender (t(49) = -2.15, p = 0.04, β = -0.29, BF = 2.86).

1. Note that models are simple linear regressions, meaning results would be equivalent even if flipping structure for time point two analysis.
2. There was a trend in the expected direction for the effect of acculturation on behavioral self-referencing in memory with CES-D included in the model as a control variable, but it did not approach significance and there was only anecdotal evidence in favor of the null hypothesis (*see below*).
